# HSF1–DBC1 axis drives prostate cancer progression by activating a metastatic transcriptional program

**DOI:** 10.1038/s12276-025-01545-7

**Published:** 2025-10-01

**Authors:** Sue Jin Moon, Hwa Jin Kim, Joung Eun Lim, Sanghoon Hong, Hong-Hee Won, Byong Chang Jeong, Jeong Hoon Kim

**Affiliations:** 1 Department of Health Sciences and Technology, Samsung Advanced Institute for Health Sciences and Technology, Sungkyunkwan University, Seoul, Republic of Korea; 2 Department of Biomedical Sciences, Metagenome Research Center, Samsung Medical Center, Seoul, Republic of Korea; 3 Department of Urology, Samsung Medical Center, Sungkyunkwan University School of Medicine, Seoul, Republic of Korea; 4 Department of Digital Health, Samsung Advanced Institute for Health Sciences and Technology, Sungkyunkwan University, Seoul, Republic of Korea

**Keywords:** Transcription, Prostate cancer, Phosphorylation, Ubiquitylation, Histone post-translational modifications

## Abstract

Heat shock factor 1 (HSF1) is a key stress-protective transcription factor that acts as a guardian of proteostasis. HSF1 also plays multifaceted roles in tumor-associated processes including proliferation and metastasis. HSF1 is frequently overexpressed and activated in a wide range of cancers, including prostate cancer, and hijacked by cancer cells to promote their survival in harsh tumor microenvironments and during metastasis. However, mechanisms underlying the persistent activation of HSF1 and its coregulators in malignancies are largely unknown. Here we show that HSF1 is highly activated and required for metastatic spread and growth of metastatic castration-resistant prostate cancer (mCRPC) cells. The HSF1-driven transcriptional program and its genome occupancy in mCRPC cells were distinct from those of castration-resistant prostate cancer cells and massively reprogrammed during the metastatic progression of castration-resistant prostate cancer cells. In addition, we report DBC1 as a key coregulator of HSF1. DBC1 positively regulated HSF1-mediated transcription and genome-wide chromatin binding of HSF1. Moreover, DBC1 was required for super-enhancer formation and activation of super-enhancer-associated HSF1 target genes, including MMP11, involved in metastasis. Mechanistically, DBC1 activated and stabilized HSF1 by enhancing trimerization and DYRK2-mediated phosphorylation of HSF1 and inhibiting CHIP-mediated HSF1 ubiquitination, thereby increasing the transcriptional activity and genome-wide binding of HSF1. Importantly, DBC1 loss suppresses the metastatic growth of mCRPC cells, and HSF1–DBC1 double-high expression correlated with worse outcomes in patients with mCRPC. Our results highlight the critical role of HSF1 as a metastasis-promoting transcription factor and a novel regulatory mechanism of HSF1 activity and stability by DBC1. Thus, targeting the HSF1–DBC1 axis could be a promising therapeutic strategy for metastatic cancers.

## HSF1 and DBC1 drive metastasis in prostate cancer

This Article explores how certain proteins help prostate cancer cells survive and spread. Prostate cancer often becomes resistant to treatment, and understanding why is crucial. Researchers focused on two proteins: HSF1 and DBC1. HSF1 helps cells manage stress, whereas DBC1 regulates other proteins. The team used lab-grown cancer cells and mice to study these proteins. They found that HSF1 is more active in advanced cancer, helping it grow and resist drugs. DBC1 supports HSF1 by stabilizing it and enhancing its activity. This partnership allows cancer cells to thrive under stress. By using techniques such as CRISPR, RNA sequencing and chromatin immunoprecipitation sequencing, they discovered that blocking HSF1 or DBC1 reduces cancer cell growth and spread. This suggests that targeting these proteins could be a new way to treat advanced prostate cancer.

This summary was initially drafted using artificial intelligence, then revised and fact-checked by the author.

## Introduction

The heat shock response (HSR) is a cytoprotective transcriptional response that helps cells adapt to various proteotoxic environmental and pathological stresses^1,2^. Heat shock factor 1 (HSF1) is a master transcriptional regulator of HSR, which plays a key role in preserving proteostasis and promoting cell survival under stress conditions by regulating the transcription of stress-responsive genes including heat shock proteins (HSPs)^2,3^. Under nonstressed, steady-state conditions, HSF1 exists mostly as an inactive monomer in the cytoplasm by forming a complex with HSP90, a negative regulator of HSF1^4^. Upon heat shock (HS), HSF1 dissociates from HSP90, becomes hyperphosphorylated, trimerizes and translocates to the nucleus, where it activates its target genes by binding to specific HSR elements (HSEs)^2,3^. In addition to maintaining proteostasis, HSF1 also plays a critical role in cancer progression. HSF1 is frequently overexpressed and associated with poor outcomes in a variety of cancers, including breast, colon and prostate cancer (PCa)^5–10^. Because cancer cells experience high levels of environmental and endogenous proteotoxic stress, including the accumulation of misfolding-prone oncoproteins, hypoxia and genotoxic stress, HSF1 is constitutively activated in cancer cells^2,3,5–7^ and drives a transcriptional program that is distinct from HSR to support malignant tumor phenotypes^8^.

PCa development and progression are dependent on androgen receptor (AR) signaling, and targeting AR with antiandrogens and androgen deprivation therapy (for example, enzalutamide and abiraterone) is a mainstay of treatment for PCa^11–14^. Although hormone therapy can improve PCa patient survival, most PCa eventually relapses and progresses to castration-resistant PCa (CRPC), which is driven by AR signaling reactivation^14,15^. AR reactivation occurs through the induction of constitutively active AR splice variants (AR-Vs) and aberrant expression of molecular chaperones (HSPs) and coregulators^13,16–18^, and AR/AR-Vs stability is highly dependent on the HSF1-mediated chaperone expression^16,18^. Approximately 90% of patients with CRPC develop metastatic CRPC (mCRPC), the leading cause of death in men worldwide^15^. However, the molecular mechanisms underlying mCRPC progression remain largely unknown. Elevated HSF1 expression is associated with higher Gleason scores and metastasis, and DTHIB, a direct HSF1 inhibitor, stimulates HSF1 degradation and inhibits xenograft tumor growth of CRPC cells^10^, suggesting HSF1 as a critical therapeutic target for mCRPC progression. Despite accumulating evidence for the role of HSF1 in cancer progression^1–3,5–7^, little is known about the coregulators and regulatory mechanisms of HSF1 in cancer cells.

Deleted in breast cancer 1 (DBC1; also known as CCAR2) functions as a coregulator of various transcription factors (TFs) including ERα, AR/AR-V7 and p53^19–26^ and as a regulator of epigenetic modifiers (for example, histone methyltransferase KMT2D/MLL4 and SUV39H1, acetyltransferases p300/CBP, deacetylases SIRT1 and HDAC3 and ubiquitin E3 ligases CHIP and MDM2)^22,24–29^. DBC1 enhances the transcriptional activity of AR by promoting its DNA binding in PCa cells^21^ and contributes to CRPC progression by activating and stabilizing AR/AR-V7^22^. Mechanistically, DBC1 inhibits the CHIP-mediated ubiquitination and degradation of AR/AR-V7. However, whether DBC1 plays a role in HSF1 signaling and mCRPC progression remains unclear. In this Article, we further explored the potential role and mechanism of DBC1 in PCa progression and report DBC1 as a critical coregulator of the HSF1-driven transcriptional program and mCRPC progression.

## Materials and methods

### Cell culture and transient transfection

All cell lines used were obtained from American Type Culture Collection or Korean Cell Line Bank and were regularly tested for mycoplasma contamination and authenticity using short tandem repeat genotyping. The cell lines were cultured in either RPMI 1640 or Dulbecco’s modified Eagle medium supplemented with 10% fetal bovine serum. A detailed description can be found in the Supplementary Information.

### Generation of mCRPC cell lines

To generate cell lines with enhanced metastatic propensity, 22RV1-LUC cells (expressing luciferase and hygromycin resistance genes) were subcutaneously injected into athymic nude mice (Orient Bio). After 3 weeks, tumor 22RV1-LUC cells (22RV1-LUC-T1) collected and purified from primary tumors were injected into the left cardiac ventricle of nude mice under ultrasound guidance (VisualSonics Vevo 2100 imaging system). Bioluminescence imaging was performed weekly to monitor metastasis formation using the IVIS Spectrum Imaging System (Xenogen, PerkinElmer). The 22RV1-LUC-T1 cells that metastasized to spinal cord (SM1), kidney (KM1) and liver (LM1) were isolated and enriched by subculturing in a medium containing hygromycin. A detailed description can be found in the Supplementary Information.

### Plasmids and antibodies

All the plasmids and antibodies used in this study are described in the Supplementary Information.

### CRISPR–Cas9-mediated knockout of HSF1 and DBC1

The 22RV1 and SM1 cells were transfected with pSpCas9(BB)-2A-GFP-sgHSF1#1 or a mixture of pSpCas9(BB)-2A-GFP-sgDBC1#1 and pSpCas9(BB)-2A-GFP-sgDBC1#3, and GFP-positive cells were sorted using a FACS cell sorter Aria III (BD Biosciences). Single colonies were isolated by limiting dilution and screened for HSF1 or DBC1 expression by immunoblot. CRISPR–Cas9-induced indel mutations were confirmed by the DNA sequencing of polymerase chain reaction (PCR) products amplified from the targeted genomic regions.

### Cell proliferation, colony formation, migration, invasion and sphere-formation assays

A detailed description can be found in the Supplementary Information.

### Xenograft experiments

Animal experiments were conducted in accordance with the principles of the Declaration of Helsinki and with the approval of the Institutional Animal Care and Use Committee of Laboratory Animal Research Center at Samsung Medical Center, ensuring the highest standards of animal welfare and ethical conduct throughout the research process. The maximum tumor size/burden (≤1,500 mm^3^) was not exceeded in our mouse experiments. Mouse xenograft experiments were performed as described above and in previous studies^20,22,23,30^. Detailed description can be found in the Supplementary Information.

### RNA-seq analysis

RNA-sequencing (RNA-seq) analysis was performed as previously described^24,30^. In brief, total RNAs were isolated using RNeasy Plus Kit (Qiagen). RNA-seq libraries were prepared using the TruSeq Stranded mRNA Library Prep Kit (Illumina) and sequenced on a NovaSeq 6000 (Illumina) platform in paired-end, 101-bp mode. A detailed description can be found in the Supplementary Information.

### ChIP-seq analysis

Chromatin immunoprecipitation sequencing (ChIP-seq) assays were performed as described previously using a SimpleChIP Plus Enzymatic Chromatin IP kit (Cell Signaling Technology) following the manufacturer’s instructions^24^. ChIP-seq libraries were constructed using the TruSeq ChIP Library Preparation Kit (Illumina) and sequenced using a NovaSeq 6000 (Illumina) to yield 70–100 million 101-bp paired-end reads per sample. A detailed description of ChIP-seq data analysis can be found in the Supplementary Information.

### qRT–PCR

A detailed description of the real-time quantitative reverse-transcription PCR (qRT–PCR) can be found in the Supplementary Information.

### Recombinant protein expression and purification

Glutathione *S*-transferase (GST)- or 6xHis-tagged recombinant proteins were expressed in *Escherichia coli* BL21(DE3) and purified using glutathione-agarose (Sigma-Aldrich) or Ni-NTA-agarose (Qiagen), respectively. HA- and 3xFLAG-tagged recombinant proteins were expressed in 293T cells and purified using HA-agarose and M2-agarose (Sigma-Aldrich), respectively, followed by elution with their corresponding peptides. In several experiments, 3xFLAG-HSF1 was purified from 293T cells after HS treatment at 42 °C for 1 h.

### Protein–protein interaction assays

Coimmunoprecipitation (CoIP) and GST pulldown assays were performed as described previously^24^. In several experiments, SM1 cell lysates and immunopurified HSF1 were treated with lambda protein phosphatase (NEB), following the manufacturer’s instructions, and used for CoIP and GST pulldown assays. A detailed description can be found in the Supplementary Information.

### Detection of phosphorylated HSF1 and kinase assays

22RV1 and SM1 cells were treated with HS at 42 °C or GSK-626616 (DYRK inhibitor, Tocris Bioscience) and lysed in FLAG lysis buffer^24^ containing phosphatase and protease inhibitor cocktails (Roche Diagnostics). HSF1 phosphorylation and protein levels were detected by immunoblot with the indicated phosphospecific antibodies. For in vivo kinase assays, 293T cells were transfected with expression plasmids for FLAG-HSF1, HA-DBC1 and HA-DYRK2 as indicated in figures. After 48-h transfection, HSF1 phosphorylation and protein levels were detected as described above. For in vitro kinase assays, the bacterially expressed and purified 6xHis-HSF1 protein was incubated with HA-DYRK2 and FLAG-DBC1 immunopurified from 293T cells in kinase buffer (25 mM Tris–HCl, pH 7.5, 10 mM MgCl_2_, 2 mM DTT, 1 mM ATP, 5 mM β-glycerophosphate and 0.1 mM Na_3_VO_4_) for 1 h at 30 °C. Phosphorylated HSF1 was detected by immunoblot.

### In vitro HSF1 cross-linking analysis

A detailed description can be found in the Supplementary Information.

### Ubiquitination assays

Ubiquitination assays were performed as described previously^22,30^. A detailed description can be found in the Supplementary Information.

### Bioinformatics and statistical analysis

Publicly available PCa gene expression datasets (GSE35988, GSE80609, GSE97284 and GSE189343) from Gene Expression Omnibus (GEO) (https://www.ncbi.nlm.nih.gov/geo) were analyzed using PRISM v5.0 for HSF1 and DBC1 mRNA expression. The correlation between HSF1 and DBC1 mRNA expression was analyzed using PRISM v5.0. A Kaplan–Meier survival analysis was performed with a publicly available CRPC dataset (GSE35988) using SigmaPlot. A pathway enrichment analysis of target genes was performed using Metascape (https://metascape.org). A gene set enrichment analysis (GSEA) was performed using GSEA program (https://www.gsea-msigdb.org/gsea/index.jsp) with target gene sets for HSF1 and DBC1. AR/AR-Vs target genes were identified by combinatorial analysis of transcriptomic datasets of CRPC cells (GSE13919, GSE99378 and GSE80743). The statistical significance was estimated using unpaired, two-tailed Student’s *t*-test. The *P* values are stated in the figure legends.

## Results

### HSF1 is overexpressed and required for the metastatic potential of mCRPC cells

**Fig. 1:**
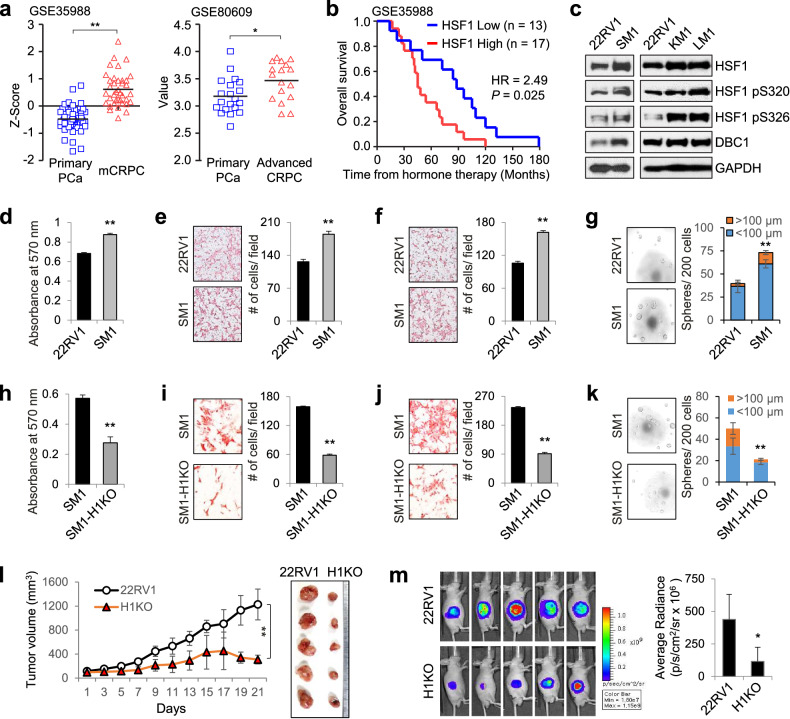
HSF1 expression is associated with mCRPC progression and is required for the tumorigenic and metastatic potential of mCRPC cells. **a** HSF1 transcript levels from two GEO datasets were determined in primary PCa and mCRPC or advanced CRPC. The statistical analysis was performed using the Student’s *t*-test. **P < 0.0001 and *P < 0.05. **b** An overall survival analysis of patients with mCRPC with low or high HSF1 expression. **c** Protein levels were monitored in 22RV1 and mCRPC cells by immunoblot using indicated antibodies. **d**–**g** The cell proliferation (**d**) migration (**e**) invasion (**f**) and sphere-formation analysis (**g**) of 22RV1 and SM1 cells. **P < 0.001. **h**–**k** The effects of H1KO on cell proliferation (**h**) migration (**i**) invasion (**j**) and sphere formation (**k**) of SM1 cells. **P < 0.001. **l** The effects of H1KO on the growth of 22RV1 xenograft tumors: tumor growth curves (left) and extracted tumors (right) are shown. 22RV1 and H1KO cells expressing luciferase (22RV1-LUC) were injected subcutaneously in male nude mice. **P < 0.001. **m** Bioluminescence images of tumor-bearing mice are shown (left), and the average signal intensity (*n* = 5) of regions of interest is quantitated (right). *P < 0.05.

To investigate the clinical significance of HSF1 in mCRPC, we first analyzed its expression and prognostic value using published PCa datasets. The expression of HSF1 was upregulated in advanced CRPC/mCRPC compared with primary PCa tumors (Fig. 1a), and patients with mCRPC with high HSF1 expression had worse survival than those with low HSF1 expression (Fig. 1b), suggesting that HSF1 expression correlates with mCRPC progression and patient outcome. To investigate the role of HSF1 in mCRPC progression, we generated mCRPC cell lines using in vivo selection following intracardiac injection of 22RV1 cells, a model cell line for CRPC (Supplementary Fig. 1). Interestingly, HSF1 protein levels were higher in mCRPC cells isolated from spine (SM1), kidney (KM1) and liver (LM1) metastatic tumors than in 22RV1 cells (Fig. 1c), indicating that HSF1 expression is increased in mCRPC cells. Because bone is the most common site of PCa metastasis, and approximately 80% of patients with CRPC develop spinal metastasis^31^, we focused on SM1 cells for further analysis and found that SM1 cells exhibited increased cell proliferation, migration, invasion and sphere-forming abilities compared with parental 22RV1 cells (Fig. 1d–g).

Next, we knocked out HSF1 in SM1 and 22RV1 cells using CRISPR–Cas9 gene editing technology (Supplementary Fig. 2a–c) and found that HSF1 knockout (H1KO) reduced the cell proliferation, migration, invasion and sphere-forming abilities of SM1 cells (Fig. 1h–k). Similar results were obtained for 22RV1 cells (Supplementary Fig. 3a–d), and H1KO caused a significant reduction in tumor volumes compared with those of control 22RV1 xenografts (Fig. 1l,m). Moreover, H1KO resensitized 22RV1 cells to enzalutamide and potentiated the cytotoxic effect of docetaxel, the first-line chemotherapy for CRPC (Supplementary Fig. 3e,f). Together, these results suggest a critical role of HSF1 in the growth, metastatic potential and drug resistance of CRPC and mCRPC cells.

### HSF1 is hyperactivated and drives a distinct transcription program in mCRPC cells

**Fig. 2:**
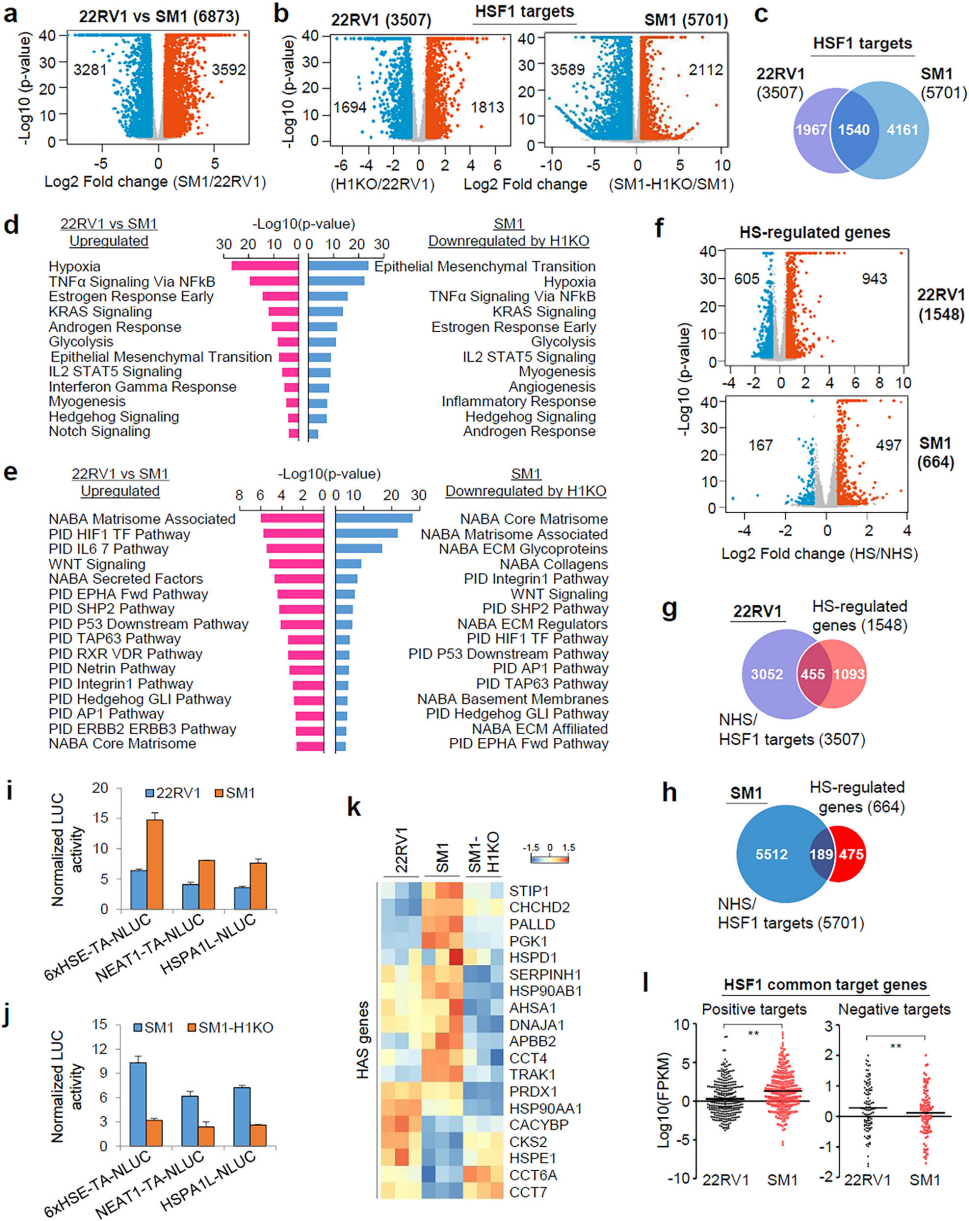
HSF1 is hyperactivated in mCRPC cells, leading to rewiring of HSF1 transcription program. **a**, **b**Volcano plots showing the differential gene expression between 22RV1 and SM1 cells (**a**) between 22RV1 and H1KO cells (**b**, left) and between SM1 and SM1-H1KO cells (**b**, right). log_2_(fold change) (log_2_FC) ≥0.58 (FC ≥1.5) and *P* < 0.05. **c** A Venn diagram showing the overlap of HSF1 target genes identified in 22RV1 and SM1 cells under NHS conditions. **d**, **e** A bar plot shows enriched hallmark (**d**) and canonical (**e**) pathways for upregulated genes in SM1 compared with 22RV1 cells and for downregulated genes by H1KO in SM1 cells. **f** Volcano plots showing the differential gene expression between NHS and HS conditions in 22RV1 and SM1 cells. **g**, **h** A Venn diagram showing the overlap between HSF1 targets and HS-regulated genes in 22RV1 (**g**) and SM1 cells (**h**). **i**, **j** 22RV1 and SM1 cells (**i**) or SM1 and SM1-H1KO cells (**j**) were transfected with indicated NanoLUC reporters, along with pRL-SV40, and dual-luciferase assays were performed. The data are means ± standard deviation (*n* = 3). **k** A heat map showing changes in HSF1 activity signature (HAS) gene expression among 22RV1, SM1 and SM1-H1KO cells. **l** Dot plots show the expression levels of positive (left) and negative (right) common HSF1 targets in 22RV1 versus SM1 cells. RNA-seq fragment per kilobase of transcript per million (FPKM) values were log-transformed for dot plots. The solid line denotes the median of each group. **P < 0.001.

To identify the HSF1-regulated genes, we performed RNA-seq experiments and found that 6,873 genes were differentially expressed between 22RV1 and SM1 cells (Fig. 2a). In addition, we found 3,507 and 5,701 HSF1 target genes in 22RV1 and SM1 cells, respectively (Fig. 2b), and only 27% (1,540/5,701) of HSF1 targets in SM1 overlapped with those in 22RV1 (Fig. 2c). A hallmark and canonical pathway analysis revealed that several metastasis- and drug resistance-associated pathways, including epithelial–mesenchymal transition, hypoxia, glycolysis, matrisome (extracellular matrix (ECM) and ECM-associated proteins) and Wnt, were upregulated in SM1 compared with 22RV1 cells and that these signaling pathways were downregulated in SM1-H1KO cells (Fig. 2d, e). These results suggest that the HSF1 transcriptional program is massively rewired or altered during metastatic progression.

Next, we identified HS-regulated genes in 22RV1 and SM1 cells (Fig. 2f). Importantly, only 13% (455/3,507) and 3.3% (189/5,701) of the HSF1 targets were HS-responsive in 22RV1 and SM1 cells, respectively (Fig. 2g–h). Gene ontology analysis showed that, although HS-regulated HSF1 targets were enriched in response to unfolded protein and cellular stresses, non-HS (NHS) HSF1 targets were enriched in biological processes related to cell–cell communication and cellular microenvironment, including cell–cell adhesion and ECM organization (Supplementary Fig. 4a). In addition, canonical pathway analysis showed that NHS HSF1 targets, but not HS targets, were enriched in matrisome-associated pathways (NABA gene sets) and that matrisome gene expression was dysregulated by H1KO (Supplementary Fig. 4b,c), indicating that HSF1 is required only for a part of the HSR and regulates distinct transcriptional programs in HS versus NHS conditions.

Interestingly, HSF1 phosphorylation levels at S320 and S326, well-established markers of HSF1 activation^32^, were higher in mCRPC cells than in 22RV1 cells (Fig. 1c). In line with these results, HSF1 reporter activities driven by 6xHSEs or HSE-containing promoters of HSPA1L and NEAT1 genes were increased in SM1 compared with 22RV1 cells (Fig. 2i) and decreased in SM1-H1KO cells (Fig. 2j). In addition, many HSF1 activity signature (HAS) genes (12/19), a 19-gene signature of HSF1 activation^33^, were upregulated in SM1 compared with 22RV1 cells, and their expression was reversed by H1KO (Fig. 2k). Moreover, among the HSF1 cotargets between 22RV1 and SM1 cells, HSF1 positive and HSF1 negative targets were upregulated and downregulated, respectively, in SM1 compared with 22RV1 cells (Fig. 2l). Together, these results suggest that HSF1 is hyperactivated and drives a distinct transcriptional program in mCRPC cells.

### Genome-wide occupancy and direct target genes of HSF1 are reprogrammed in mCRPC cells

**Fig. 3:**
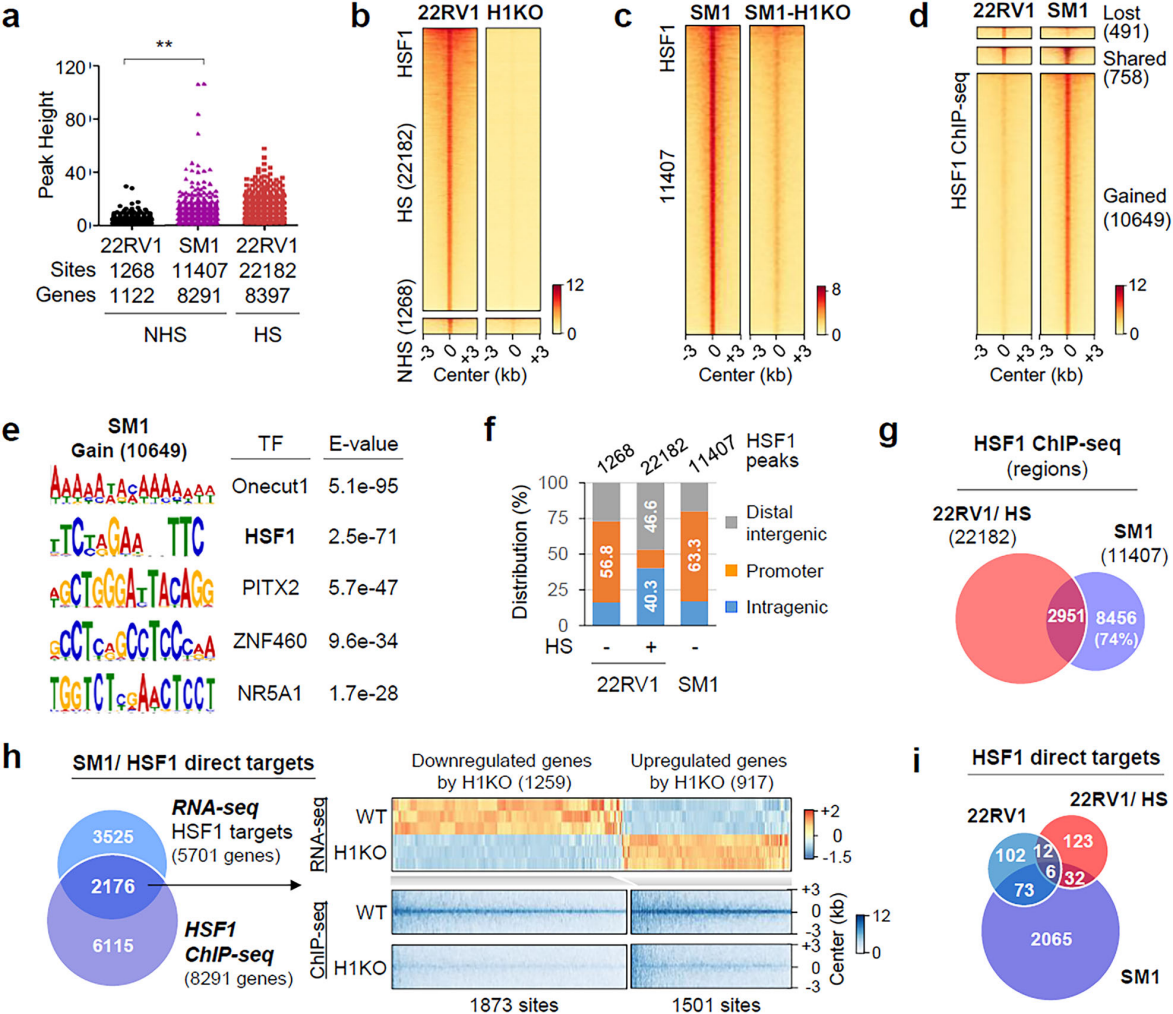
HSF1 chromatin occupancy and direct targets are reprogrammed in mCRPC cells. **a** ChIP-seq peak heights for each HSF1 binding site in 22RV1 under NHS and HS conditions and SM1 cells under NHS conditions. **P < 0.001. **b**, **c** Heat maps of HSF1 ChIP-seq signals in 22RV1 versus H1KO (**b**) under NHS and HS conditions and SM1 versus SM1-H1KO (**c**) under NHS conditions. The signals within 3 kb around the center of HSF1 ChIP-seq peaks are ordered by the decreasing ChIP-seq signal in SM1 cells. **d** Heat maps showing changes in ChIP-seq signals of HSF1 in 22RV1 versus SM1 cells. **e** A MEME-ChIP motif analysis reveals the top enriched motifs in HSF1 binding sites in SM cells. **f** The genomic distribution of HSF1 ChIP-seq peak regions in 22RV1 (NHS and HS) and SM1 (NHS) cells. The promoter regions were defined as regions ±1 kb around transcription start sites. **g** A Venn diagram showing the genome-wide overlap of HSF1 ChIP-seq peaks identified in 22RV1 cells under HS conditions and SM1 cells under NHS conditions. **h** A Venn diagram showing the overlap of HSF1-regulated genes (RNA-seq) and HSF1-bound genes (ChIP-seq) in SM1 cells. The heat maps show changes in HSF1 direct target gene expression and HSF1 ChIP-seq signals in SM1 versus SM1-H1KO. **i** A Venn diagram showing the overlap of HSF1 direct target genes between SM1 and 22RV1 (NHS and HS) cells.

Next, we performed ChIP-seq to identify genome-wide HSF1 binding sites (Supplementary Fig. 5a). HSF1 showed robust chromatin binding and increased binding strength in SM1 compared with 22RV1 cells (Fig. 3a–c). A differential peak analysis revealed that 491 and 10,649 HSF1 binding sites were lost and gained, respectively, in SM1 compared with 22RV1 cells (Fig. 3d). In motif analysis, the gained HSF1 binding sites were highly enriched for canonical HSE (Fig. 3e), and HSE enrichment was greatly increased in SM1 compared with 22RV1 (Supplementary Fig. 5b). Consistent with previous studies^8,34,35^, HSF1 chromatin binding was dramatically increased by HS (Fig. 3a,b). HSF1 was predominantly enriched in promoters under NHS conditions, whereas HSF1 was mainly found in distal intergenic and intragenic regions under HS conditions (Fig. 3f). In addition, 74% of the HSF1 binding sites in SM1 did not overlap with those in 22RV1 cells, even after HS (Fig. 3g and Supplementary Fig. 5c). These results indicate the substantial reprogramming of the HSF1 cistrome during metastasis.

Next, we integrated HSF1 ChIP-seq data with transcriptomic data to identify direct targets of HSF1. Among the 5,701 HSF1-regulated genes in SM1 cells, 2,176 genes (38.2%) were direct targets (Figs. 3h), and 5.5% (193/3,507) and 37.7% (173/459) of NHS- and HS-regulated HSF1 targets, respectively, were direct targets in 22RV1 cells (Supplementary Fig. 5d,e). Interestingly, only 79 and 38 genes were HSF1 direct cotargets between SM1 and either NHS or HS-treated 22RV1 cells, respectively (Fig. 3i), indicating that HSF1 direct targets are reprogrammed during metastatic progression.

### DBC1 functions as a coregulator for HSF1

**Fig. 4:**
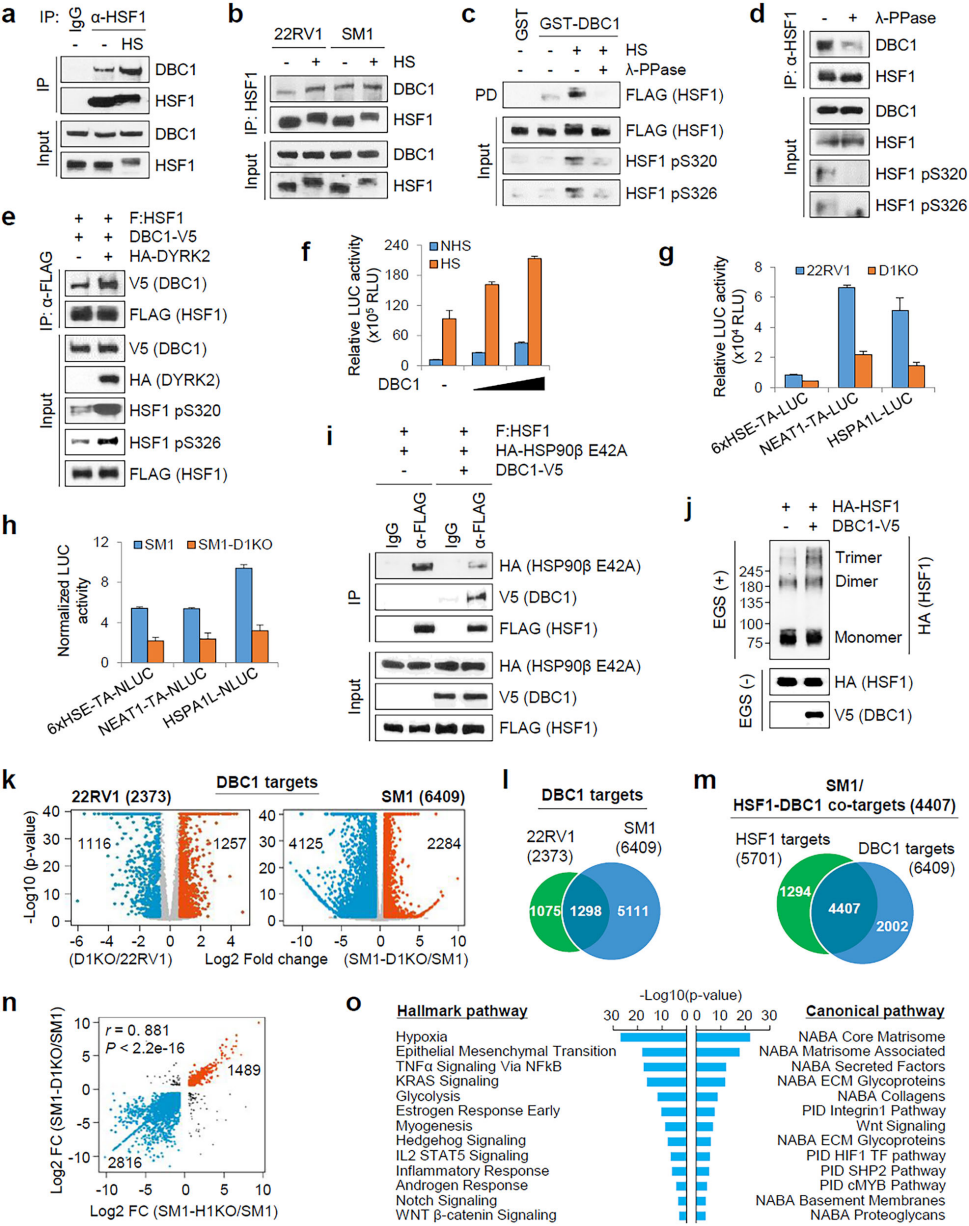
Coregulator function of DBC1 for HSF1 and requirement of DBC1 for transcriptional regulation of genes involved in mCRPC progression. **a** The 22RV1 cells were treated with or without HS at 42 °C for 1 h. The cell lysates were immunoprecipitated with normal IgG or an anti-HSF1 antibody. The input and immunoprecipitated proteins were analyzed by immunoblot with indicated antibodies. **b** Endogenous CoIP experiments were performed as described in **a** in 22RV1 and SM1 cells. **c** A pulldown of FLAG-HSF1 untreated or treated with λ-phosphatase with GST-DBC1. The bound and input proteins were analyzed by immunoblot with indicated antibodies. **d** SM1 cell extracts were treated with or without λ-phosphatase and immunoprecipitated with anti-HSF1 antibody. The input and immunoprecipitated proteins were analyzed by immunoblot with indicated antibodies. **e** The 293T cells were transfected with the indicated constructs, and the cell lysates were immunoprecipitated with FLAG-M2 agarose beads. The input and immunoprecipitated proteins were analyzed by immunoblot with indicated antibodies. **f** The 22RV1 cells were transfected with 6xHSE-LUC reporter and an increasing amount of HA-DBC1 vector and collected for luciferase assays. The data are means ± standard deviation (s.d.) (*n* = 3). **g** 22RV1 and D1KO cells were transfected with indicated reporters and collected for luciferase assays. The data are means ± s.d. (*n* = 3). **h** SM1 and SM1-D1KO cells were transfected with indicated NanoLUC reporters, along with pRL-SV40, and collected for dual-luciferase assays. The data are means ± s.d. (*n* = 3). **i** The 293T cells were transfected as indicated, and FLAG-HSF1 immunoprecipitates were analyzed by immunoblot with the indicated antibodies. To strengthen the transient interaction between HSF1 and HSP90β, the client-trapping mutant of HSP90β (HSP90β E42A) was used. **j** Cell extracts from 293T cells transfected with the indicated expression plasmids were subjected to in vitro cross-linking with 2 mM ethylene glycol bis(succinimidyl succinate) (EGS) and analyzed by immunoblot with the indicated antibodies. **k** Volcano plots showing the differential gene expression between 22RV1 and D1KO cells and between SM1 and SM1-D1KO cells. log_2_(fold change) (log_2_FC) ≥0.58 (FC ≥1.5) and *P* < 0.05. **l** A Venn diagram showing the overlap of DBC1 target genes identified in 22RV1 and SM1 cells. **m** A Venn diagram showing the overlap between HSF1 and DBC1 target genes in SM1 cells. **n** Scatter plots of Pearson correlation between log_2_FC in expression levels of HSF1–DBC1 cotarget genes in SM1 cells upon H1KO and D1KO. **o** A bar plot shows the enriched hallmark and canonical pathways for HSF1–DBC1 positive cotarget genes in SM1 cells.

Because DBC1 acts as a tumor-promoting coregulator in CRPC cells^22^, we investigated whether DBC1 functions as a coregulator of HSF1. In CoIP experiments, endogenous HSF1 bound weakly to DBC1 in 22RV1 cells, and this interaction was increased by HS (Fig. 4a), suggesting that stress-induced HSF1 activation enhances its interaction with DBC1. Interestingly, the HSF1–DBC1 interaction was increased in SM1 compared with 22RV1 cells even in the absence of HS (Fig. 4b). In GST pulldown assays using FLAG-HSF1 purified from 293T cells treated with HS, HS treatment increased HSF1 phosphorylation and its interaction with DBC1, and HSF1 dephosphorylation by λ-phosphatase almost eliminated the HSF1–DBC1 interaction (Fig. 4c). CoIP experiments using SM1 lysates treated with λ-phosphatase confirmed the phosphorylation-dependent interaction of HSF1 with DBC1 (Fig. 4d). In addition, the overexpression of DYRK2, which phosphorylates HSF1 at S320 and S326^32^, increased the HSF1–DBC1 interaction (Fig. 4e), and HSF1 phosphomimetic mutant (S320D/S326D) showed stronger transcriptional activity and higher binding affinity for DBC1 compared with wild-type HSF1 (Supplementary Fig. 6a,b), suggesting that activating phosphorylation of HSF1 is required for its binding to DBC1.

Next, we knocked out DBC1 in 22RV1 and SM1 cells (Supplementary Fig. 2d–f) and performed HSF1 reporter assays. DBC1 overexpression enhanced both basal and HS-induced HSF1 activity in a dose-dependent manner in 22RV1 cells (Fig. 4f), and by contrast, DBC1 knockout (D1KO) reduced HSF1 activity in 22RV1 and SM1 cells (Fig. 4g, h), suggesting that DBC1 is required for the transcriptional activity of HSF1. Trimerization is required for the efficient DNA binding and transcriptional activity of HSF1, and HSP90 negatively regulates HSF1 activity by monomerizing HSF1 trimers^2,4,5^. Intriguingly, the interaction of HSF1 with HSP90 decreased when DBC1 was coexpressed in CoIP experiments (Fig. 4i). In line with these results, HSF1 trimerization was increased by DBC1 overexpression (Fig. 4j), suggesting that DBC1 as a coregulator promotes HSF1-mediated transcription by enhancing HSF1 trimerization through blocking the interaction between HSF1 and HSP90.

We then performed RNA-seq analysis to investigate the role of DBC1 in the HSF1-driven gene expression program and identified 2,373 and 6,409 DBC1 target genes in 22RV1 and SM1 cells, respectively (Fig. 4k). Only 20.3% (1,298/6,409) of DBC1 targets in SM1 overlapped with those in 22RV1 cells (Fig. 4l), suggesting that the DBC1-mediated transcriptional program was largely altered in SM1 cells. Importantly, 77.3% (4,407/5,701) of HSF1 targets overlapped with DBC1 targets in SM1 cells (Fig. 4m and Supplementary Fig. 6c), but only 30% (1,052/3,507) were overlapping genes in 22RV1 cells (Supplementary Fig. 6d). Directions of changes in HSF1–DBC1 cotarget gene expression caused by H1KO and D1KO were positively correlated in SM1 cells (Fig. 4n), indicating that DBC1 acts as a coactivator and corepressor of HSF1-mediated transactivation and transrepression, respectively. Moreover, HSF1–DBC1 positive cotargets were enriched in hypoxia, epithelial–mesenchymal transition, glycolysis, Wnt and matrisome-associated pathways (Fig. 4o and Supplementary Fig. 6e), suggesting that HSF1 and DBC1 cooperate to regulate a common transcriptional program that is critical for mCRPC progression.

AR signaling continues to play a critical role in all stages of PCa including mCRPC, and both HSF1 and DBC1 play a critical role in fueling AR/AR-V7 signaling in CRPC cells^10,22,36^. Indeed, HSF1–DBC1 positive cotargets were also enriched in the androgen-response pathway (Fig. 4o). In addition, comparative analysis of transcriptomic data showed that 15.8% (695/4,407) of HSF1–DBC1 cotargets in SM1 cells overlapped with AR/AR-V7-regulated genes, but the majority (84.2%, 3,712/4,407) of HSF1–DBC1 cotargets were independent of AR/AR-V7-mediated transcriptional program (Supplementary Fig. 6f). A canonical pathway analysis revealed that the AR/AR-V7-independent HSF1–DBC1 cotarget genes were highly enriched in pathways associated with cancer metastasis, including matrisome-related (NABA gene sets), Fanconi, PLK1 and Wnt signaling pathways, compared with HSF1–DBC1-AR/AR-V7 common targets (Supplementary Fig. 6g). Of the AR/AR-V7-independent HSF1–DBC1 targets, we selected the MMP11 gene, which has been shown to be a critical mediator and biomarker of CRPC progression and metastasis^37,38^, for validation. H1KO and D1KO resulted in a decrease in both mRNA and protein levels of MMP11 (Supplementary Fig. 7a,b). Similar results were observed in AR-negative PC3 cells depleted of HSF1 or DBC1 (Supplementary Fig. 7c,d). These results suggest that the role of the HSF1–DBC1 axis in mCRPC progression is not restricted to the regulation of AR/AR-V7 signaling pathway but includes important functions in the regulation of pathways associated with matrisome remodeling and metastasis.

### DBC1 plays a critical role in genome-wide loading of HSF1 and HSF1 direct target gene expression

**Fig. 5:**
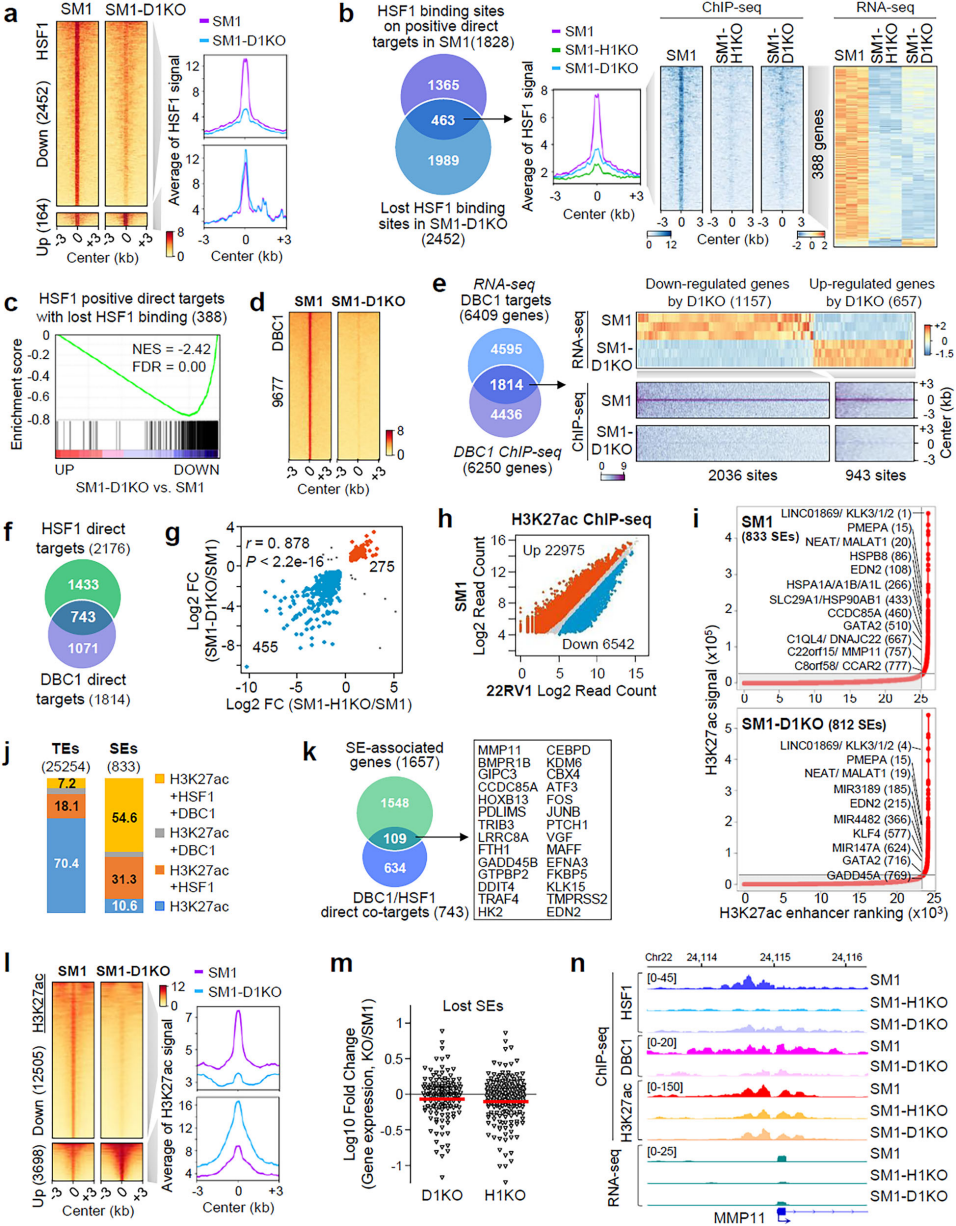
DBC1 is required for efficient chromatin binding of HSF1 and SE-associated HSF1 target gene expression. **a** Left: heat maps of downregulated and upregulated HSF1 ChIP-seq signals in SM1 versus SM1-D1KO cells. Right: plots of the average HSF1 ChIP-seq signals at downregulated and upregulated regions. **b** Left: a Venn diagram showing HSF1 positive direct targets with lost HSF1 binding in SM1 versus SM1-D1KO cells. Middle: a plot of the average HSF1 ChIP-seq signals and heat maps of HSF1 signals on HSF1 positive direct targets with lost HSF1 binding in SM1 versus SM1-H1KO or SM1-D1KO cells. Right: heat maps showing changes in the expression of HSF1 positive direct targets with lost HSF1 binding in SM1 versus SM1-H1KO or SM1-D1KO cells. **c** A GSEA showing the negative enrichment of HSF1 positive direct targets with lost HSF1 binding in SM1-D1KO compared with SM1 cells. **d** Heat maps of DBC1 ChIP-seq signals in SM1 versus SM1-D1KO cells. **e** Left: a Venn diagram showing the overlap of DBC1-regulated genes and DBC1-bound genes in SM1 cells. Right: heat maps showing changes in DBC1 direct target gene expression and DBC1 ChIP-seq signals in SM1 versus SM1-D1KO cells. **f** A Venn diagram showing HSF1–DBC1 direct cotargets in SM1 cells. **g** Scatter plots of a Pearson correlation between log_2_(fold change) (log_2_FC) in expression levels of HSF1–DBC1 direct cotargets in SM1 cells upon H1KO and D1KO. **h** Scatter plots showing changes in H3K27ac ChIP-seq signals in 22RV1 versus SM1 cells. **i** Ranked plots of SEs and TEs defined on the basis of H3K27ac ChIP-seq in SM1 and SM1-D1KO cells. **j** Stacked bar charts showing the overlap of H3K27ac-defined TEs and SEs with HSF1 and/or DBC1. **k** A Venn diagram showing the overlap of SE-associated genes with DBC1-HSF1 direct cotargets in SM1 cells. SE-associated HSF1–DBC1 direct cotargets include many metastasis-associated genes, which are listed in the box. **l** Left: heat maps of downregulated and upregulated H3K27ac ChIP-seq signals in SM1 versus SM1-D1KO cells. Right: plots of average H3K27ac signals at downregulated and upregulated regions. **m** Dot plots showing log_10_FCs in gene expression that are regulated by lost SEs in SM1 cells upon D1KO or H1KO. The red lines indicate the median FC values. **n** Snapshots of ChIP-seq tracks for HSF1, DBC1 and H3K27ac and RNA-seq tracks at the MMP11 SE in SM1, SM1-H1KO and SM1-D1KO cells.

To investigate the role of DBC1 in the genome-wide chromatin binding of HSF1, we performed HSF1 ChIP-seq in SM1-D1KO cells; 21.5% (2,452/11,407) of HSF1 peaks were lost in SM1-D1KO compared with SM1 cells, whereas only 1.4% of HSF1 peaks were gained (Fig. 5a and Supplementary Fig. 6h). Similar results were observed in 22RV1 cells (Supplementary Fig. 6i). In addition, 25.3% (463/1,828) of HSF1 peaks on positive direct targets of HSF1 were lost by D1KO in SM1 cells (Fig. 5b), and the expression of the majority of HSF1 positive direct targets with lost HSF1 binding was downregulated in SM1-D1KO cells (Fig. 5b, c), suggesting a critical role of DBC1 in genome-wide HSF1 binding and HSF1 direct target gene expression.

To identify the direct targets of DBC1, we performed ChIP-seq for DBC1 and identified 9,677 DBC1 peaks in SM1 cells (Fig. 5d). The integration of ChIP-seq and RNA-seq data showed that, among 6,409 DBC1-regulated genes, 1,814 genes (28.3%) were direct targets with DBC1-bound sites (Fig. 5e), indicating that DBC1 can regulate gene expression via direct and indirect mechanisms. An integrative ChIP-seq analysis showed that 19.2% (1,860/9,677) of DBC1-binding sites were cooccupied with HSF1 (Supplementary Fig. 6j). In addition, 41% (743/1,814) of DBC1 direct targets overlapped with HSF1 direct targets (Fig. 5f). Importantly, the direction of changes in HSF1–DBC1 direct cotarget gene expression caused by H1KO and D1KO was positively correlated (Fig. 5g), indicating again that DBC1 regulates HSF1-mediated transactivation and transrepression by acting as a coregulator.

### DBC1 plays an important role in SE formation and SE-associated HSF1 target gene expression

DBC1 plays a critical role in the formation and function of super-enhancers (SEs), clusters of enhancers densely loaded with TFs, coregulators and enhancer marks such as acetylated histone H3 lysine 27 (H3K27ac)^24^. To investigate the nature of HSF1- and DBC1-bound sites and SE dynamics during cancer progression, we profiled active chromatin regions using H3K27ac ChIP-seq (Supplementary Fig. 8a) and found a massive remodeling of active chromatin regions, with 22,975 upregulated and 6,542 downregulated H3K27ac regions, in SM1 compared with 22RV1 cells (Fig. 5h and Supplementary Fig. 8b). A rank ordering of SE analysis identified 1,220 SEs/23,106 typical enhancers (TEs) in 22RV1 and 833 SEs/25,254 TEs in SM1 cells (Fig. 5i and Supplementary Fig. 8c), of which, 589 and 175 SEs and 5,643 and 7,575 TEs were lost and gained in SM1 cells (Supplementary Fig. 8d), indicating substantial changes in the SE/TE landscapes of SM1 cells.

An integrative analysis of ChIP-seq data in SM1 cells revealed that, whereas HSF1 and DBC1 were coenriched at only 7.2% of TEs, 54.6% (455/833) of SEs were coenriched for HSF1 and DBC1 (Fig. 5j and Supplementary Fig. 8e). Interestingly, 14.7% (109/743) of HSF1–DBC1 direct cotarget genes were associated with SEs, including many metastasis-related genes (Fig. 5k). D1KO and H1KO resulted in genome-wide dysregulation of H3K27ac (Fig. 5l and Supplementary Fig. 8g) and, accordingly, impaired SE/TE landscapes (Fig. 5i and Supplementary Fig. 8c,f,h). Importantly, the expression of lost SE-associated genes was downregulated in SM1-D1KO and SM1-H1KO cells (Fig. 5m). Genome browser snapshots showed a broad co-occupancy of HSF1, DBC1 and H3K27ac at SEs of MMP11, BMPR1B and GIPC3 genes, and their occupancy and target gene transcription were reduced by D1KO (Fig. 5n and Supplementary Fig. 8i,j). Together, these results suggest a critical role for DBC1 in regulating H3K27ac and TE/SE dynamics and promoting the transcriptional activation of SE-associated HSF1 target genes.

### DBC1 positively regulates HSF1 stability by promoting HSF1 phosphorylation and inhibiting HSF1 ubiquitination

**Fig. 6:**
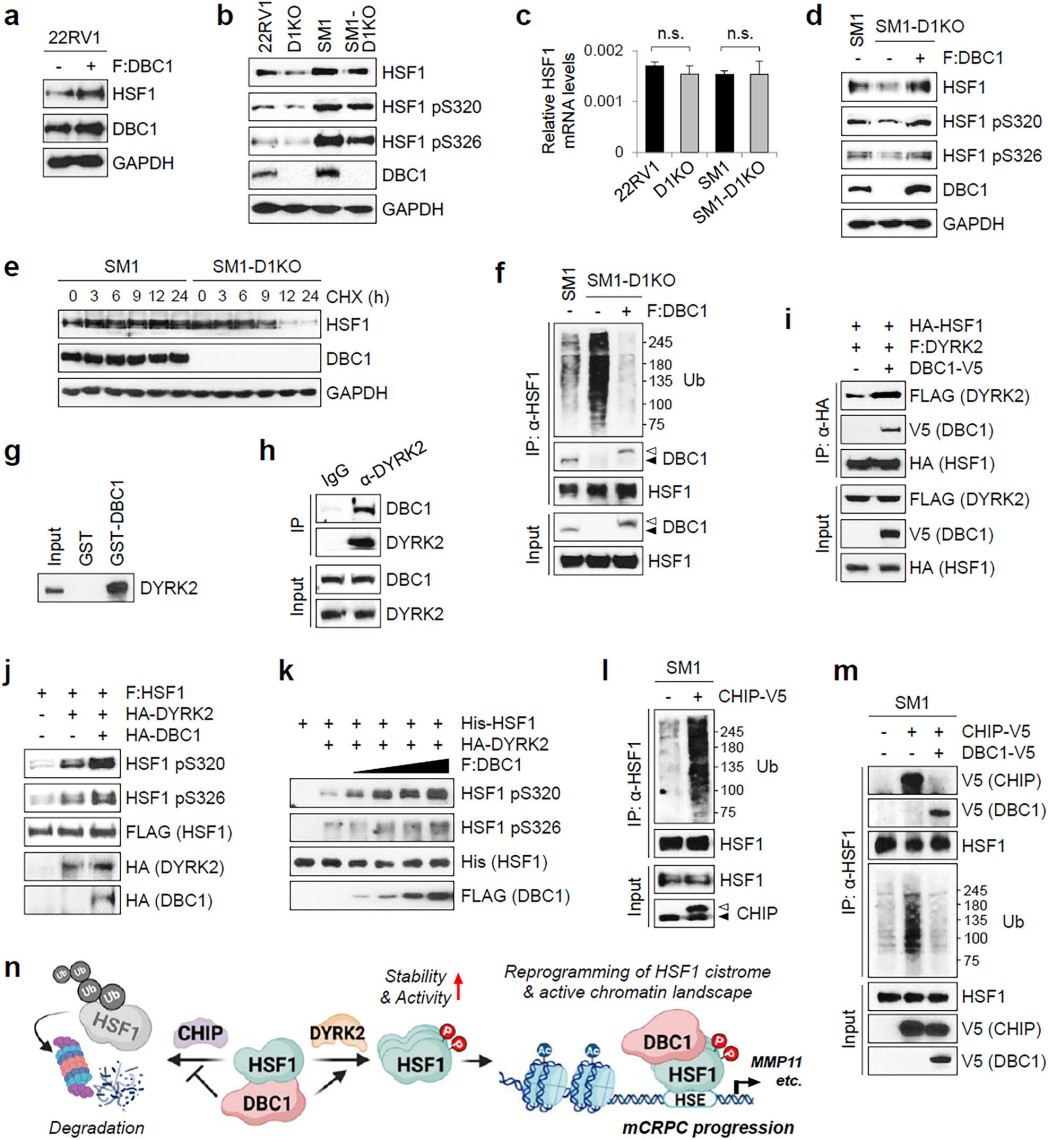
DBC1 regulates HSF1 stability by promoting DYRK2-mediated HSF1 phosphorylation and inhibiting CHIP-mediated HSF1 ubiquitination. **a** The 22RV1 cell lysates transfected with empty or FLAG-DBC1 expression vector were immunoblotted with the indicated antibodies. **b** Cell lysates of 22RV1, SM1 and their D1KO counterparts were analyzed by immunoblot with indicated antibodies. **c** mRNA levels of HSF1 were analyzed by qRT–PCR in 22RV1, D1KO, SM1 and SM1-D1KO cells. The data are means ± standard deviation (*n* = 3). n.s., not significant (*P* > 0.05). **d** The expression levels of indicated proteins were analyzed by immunoblot in SM1, SM1-D1KO and 4xFLAG-DBC1-expressing lentivirus-infected SM1-D1KO cells. **e** SM1 and SM1-D1KO cells were treated with 50 µg/ml cycloheximide (CHX) and collected at the indicated time points. Cell lysates were analyzed by immunoblot with the indicated antibodies. **f** Cell lysates of SM1, SM1-D1KO and 4xFLAG-DBC1-expressing lentivirus-infected SM1-D1KO cells were immunoprecipitated with anti-HSF1 antibody and immunoblotted with indicated antibodies. The closed and open arrowheads indicate endogenous and 4xFLAG-tagged DBC1 proteins, respectively. **g** In vitro-translated HA-DYRK2 was incubated with recombinant GST-DBC1. The bound proteins were analyzed by immunoblot with anti-HA antibody. **h** The endogenous CoIP between DBC1 and DYRK2 in SM1 cells. Immunoprecipitation and immunoblot were performed using the indicated antibodies. **i** The 293T cell extracts transfected with expression vectors as indicated were immunoprecipitated and immunoblotted with the indicated antibodies. **j** The 293T cell extracts transfected with expression vectors as indicated were analyzed by immunoblot using the indicated antibodies. **k** In vitro kinase assays using recombinant His-HSF1, immunopurified HA-DYRK2 and FLAG-DBC1 as indicated. The reactions were analyzed by immunoblot using the indicated antibodies. **l** SM1 cell lysates transfected with an empty or CHIP-V5 expression vector were immunoprecipitated with anti-HSF1 antibody and immunoblotted with the indicated antibodies. The closed and open arrowheads indicate endogenous and V5-tagged CHIP proteins, respectively. **m** SM1 cell lysates transfected with the indicated expression vectors were immunoprecipitated with anti-HSF1 antibodies and immunoblotted with the indicated antibodies. **n** The role of DBC1 as a HSF1 coregulator in mCRPC progression. DBC1 inhibits CHIP-mediated HSF1 ubiquitination and enhances HSF1 trimerization and DYRK2-mediated HSF1 phosphorylation, thereby increasing HSF1 stability and activity. This coregulator activity of DBC1 contributes to the reprogramming of HSF1 cistrome and the active chromatin landscape, including SEs, and thus promotes mCRPC progression by activating metastasis-associated genes, including MMP11. **n** was created using bioRender.com.

Next, we investigated the mechanism by which DBC1 regulates HSF1 activity. mCRPC cells expressed higher levels of DBC1 than 22RV1 cells (Fig. 1c), and DBC1 overexpression increased HSF1 levels in 22RV1 cells (Fig. 6a), suggesting a link between HSF1 and DBC1 protein levels. Whereas neither HSF1 overexpression nor H1KO altered mRNA and protein levels of DBC1 in 22RV1 and SM1 cells (Supplementary Fig. 9a–d), D1KO decreased the protein and activating phosphorylation levels of HSF1 without affecting its mRNA levels (Fig. 6b, c). In addition, the reexpression of DBC1 in SM1-D1KO cells rescued the protein and activating phosphorylation levels of HSF1 (Fig. 6d), indicating that DBC1 regulates HSF1 expression at the posttranslational level. When SM1 and 22RV1 cells were treated with cycloheximide, D1KO caused a faster degradation of HSF1 (Fig. 6e and Supplementary Fig. 9e). D1KO increased ubiquitination levels of HSF1 in SM1 and 22RV1 cells (Fig. 6f and Supplementary Fig. 9f, g), and the reexpression of DBC1 in SM1-D1KO cells dramatically reduced ubiquitination levels of HSF1 (Fig. 6f), suggesting that DBC1 positively regulates HSF1 stability by inhibiting HSF1 ubiquitination.

DYRK2-mediated HSF1 phosphorylation at S320/S326 has been shown to increase HSF1 protein levels^32^. GSK-626616, a pan-DYRK inhibitor, reduced HSF1 protein and S320/S326 phosphorylation levels and increased HSF1 ubiquitination (Supplementary Fig. 9h, i). DYRK2 depletion also reduced HSF1 protein and phosphorylation levels (Supplementary Fig. 9j). Next, we investigated the role of DBC1 in DYRK2-mediated HSF1 phosphorylation. The GST pulldown and endogenous CoIP experiments showed that DBC1 interacts with DYRK2 (Fig. 6g, h). When overexpressed in 293T cells, DBC1 increased the interaction between HSF1 and DYRK2 (Fig. 6i) and DYRK2-mediated HSF1-S320/S326 phosphorylation (Fig. 6j). In vitro kinase assays confirmed the enhancement of DYRK2-mediated HSF1 phosphorylation by DBC1 (Fig. 6k), suggesting that DBC1 enhances HSF1-S320/S326 phosphorylation by facilitating the HSF1–DYRK2 association, thereby promoting HSF1 stability.

Although HSF1 turnover is tightly regulated by the ubiquitin–proteasome pathway^39^, only a few E3 ligases for HSF1, including FBXW7 and NEDD4, have been identified to ubiquitinate HSF1 and promote its degradation^40,41^. CHIP is a dual-function cochaperone/ubiquitin E3 ligase that plays a critical role in protein quality control^42^. Numerous studies have shown that CHIP acts as a tumor suppressor in CRPC by mediating the ubiquitination and degradation of oncogenic TFs such as AR/AR-V7 and HIF1α^22,42–44^. We thus tested whether CHIP acts as an E3 ligase for HSF1. CHIP overexpression resulted in a dramatic increase in HSF1 ubiquitination in SM1 cells (Fig. 6l). Given our previous findings that DBC1 inhibits the E3 ligase activity of CHIP in CRPC cells^22^, we tested the effect of DBC1 on CHIP-mediated HSF1 ubiquitination. In CoIP experiments, CHIP bound to endogenous HSF1 in SM1 cells, and DBC1 overexpression blocked the interaction between HSF1 and CHIP and inhibited CHIP-mediated HSF1 ubiquitination (Fig. 6m). Together, these results suggest that DBC1 contributes to HSF1 stability by promoting HSF1-S320/S326 phosphorylation through enhancing the HSF1–DYRK2 association and by inhibiting CHIP-mediated HSF1 ubiquitination through blocking the HSF1–CHIP interaction (Fig. 6n).

### DBC1 and HSF1 are required for the metastatic spread and growth of mCRPC cells and associated with mCRPC progression

**Fig. 7:**
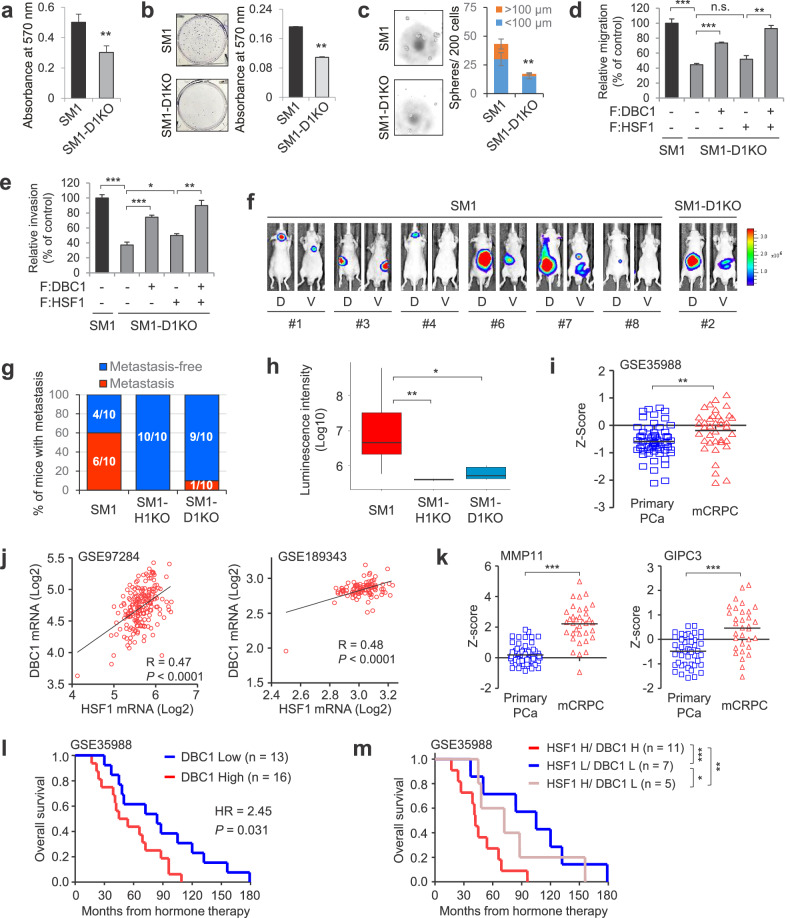
DBC1 is required for the metastatic spread and growth of mCRPC cells and is associated with mCRPC progression. **a**–**c** The cell proliferation (**a**) colony formation (**b**) and prostasphere-formation analyses (**c**) of SM1 and SM1-D1KO cells. **P < 0.001. **d**, **e** Transwell migration (**d**) and invasion analysis (**e**) of SM1 and SM1-D1KO cells transfected with FLAG-HSF1 or/and FLAG-DBC1 expression vector. ***P < 0.0001, **P < 0.001 and *P < 0.01. n.s., not significant. **f**–**h** Bioluminescence images of metastatic tumor-bearing mice are shown (**f**); the percentage of mice with metastasis is indicated from SM1, SM1-H1KO and SM1-D1KO groups (*n* = 10 per group) (**g**) and the average signal intensity (*n* = 10) of regions of interest is quantitated (**h**). The boxes represent the 25th–75th percentile range. Wilcoxon two-tailed rank-sum test at day 78, **P < 0.001 and *P < 0.05. **i** The DBC1 transcript levels from the GSE35988 dataset were determined in primary PCa and mCRPC. **P < 0.001. **j** A correlation analysis of HSF1 and DBC1 expression in mCRPC. Plotted data are log_2_ mRNA expression from GSE97284 (*n* = 188) and GSE189343 (*n* = 103) datasets. **k** The transcript levels of MMP11 and GIPC3 from the GSE35988 dataset were determined in primary PCa and mCRPC. ***P < 0.0001. **l**, **m** The overall survival analysis of patients with mCRPC with high or low DBC1 expression (**l**) and with different HSF1 and DBC1 expression levels (**m**). ***P = 0.007, **P = 0.086 and *P = 0.473.

We examined the D1KO effect on the metastatic properties of mCRPC cells. D1KO attenuated the cell proliferation, colony and sphere formation in SM1 cells (Fig. 7a–c) and inhibited their migration and invasion abilities (Fig. 7d, e and Supplementary Fig. 10a, b). Similar D1KO effects were observed in 22RV1 cells (Supplementary Fig. 10c–g), indicating that DBC1 is required for the metastatic ability of CRPC and mCRPC cells. In fact, the reexpression of DBC1 in SM1-D1KO cells efficiently rescued HSF1 protein levels and cell migration/invasion (Fig. 7d, e and Supplementary Fig. 10h, i). To investigate the role of DBC1 in the HSF1-mediated metastatic phenotype of mCRPC cells, we overexpressed HSF1 to recover the reduced protein level of HSF1 caused by D1KO in SM1 cells. Strikingly, the overexpression of HSF1 failed to efficiently reverse the defects in migration and invasion in SM1-D1KO cells (Fig. 7d, e and Supplementary Fig. 10h, i), suggesting a requirement of DBC1 for the HSF1-mediated metastatic behavior of SM1 cells. Indeed, the coexpression of HSF1 with DBC1 almost completely rescued cell migration and invasion (Fig. 7d, e and Supplementary Fig. 10h, i). Similar rescue patterns were observed for the expression of MMP11, a HSF1–DBC1 cotarget gene (Supplementary Fig. 10h, j). These results confirm that both HSF1 and DBC1 are required for the metastatic behavior of mCRPC cells and indicate that DBC1 is required not just for HSF1 stability but also for HSF1 activity as a transcriptional coregulator.

Given that MMP11 serves as a critical regulator of PCa metastasis^37,38^ and is one of the direct targets of HSF1–DBC1 axis (Fig. 5n and Supplementary Fig. 7a–d), we next assessed the role of MMP11 in the metastatic properties of mCRPC cells. The depletion of MMP11 resulted in a reduced migration and invasion of SM1 cells (Supplementary Fig. 7e, f). Importantly, the restoration of MMP11 expression in SM1-H1KO and SM1-D1KO cells significantly rescued the defects in cell migration and invasion (Supplementary Fig. 7g–j), indicating that MMP11 is a key downstream target gene of the HSF1–DBC1 axis for promoting the migration and invasion of mCRPC cells.

We next examined the effect of H1KO and D1KO on the metastatic capacity of SM1 cells in a mouse metastasis model (Supplementary Fig. 10k). An intracardiac injection of SM1 cells resulted in metastasis to multiple organs (for example, kidney, liver, bone and lymph nodes) in 60% of the mice (Fig. 7f–h and Supplementary Fig. 10l). Strikingly, metastasis was completely abolished by H1KO and almost completely blocked by D1KO (Fig. 7f–h and Supplementary Fig. 10l), indicating the critical roles of HSF1 and DBC1 in the metastatic spread and growth of mCRPC cells.

Finally, we investigated the clinical significance of the HSF1–DBC1 axis in mCRPC. A higher DBC1 expression was observed in patients with mCRPC than in patients with primary PCa (Fig. 7i). In addition, in line with previous reports showing the gain of whole chromosome 8, in which HSF1 and DBC1 genes are localized, during PCa progression^45,46^, HSF1 and DBC1 mRNA levels were modestly but significantly correlated in mCRPC cohorts (Fig. 7j). Consistent with the upregulation of HSF1 and DBC1 in mCRPC (Figs. 1a and 7i), metastasis-associated HSF1–DBC1 direct cotarget genes were also upregulated in mCRPC compared with patients with primary PCa (Fig. 7k and Supplementary Fig. 11). Moreover, patients with mCRPC with high DBC1 expression showed worse outcomes than those with low DBC1 expression (Fig. 7l). Importantly, HSF1–DBC1 double-high patients had worse overall survival compared with HSF1–DBC1 double-low patients, and there was a trend that, among patients with HSF1-high mCRPC, high DBC1 expression predicted worse prognosis than low DBC1 expression (Fig. 7m), suggesting an association between DBC1 expression and poor mCRPC prognosis and a promoting role of the HSF1–DBC1 axis in mCRPC progression.

## Discussion

Mounting evidence has revealed that HSF1 plays multifaceted roles in tumor-associated cellular processes including cell proliferation, tumor microenvironment (TME) remodeling, chemoresistance and metastasis^5,6,8^. HSF1 is chronically activated in a wide range of cancers and hijacked by cancer cells to promote their survival and malignant properties in harsh TMEs^47^. Thus, defining the role of HSF1 is essential for understanding cancer progression and developing novel cancer therapeutics. Here, we added another line of evidence supporting the role of HSF1 as a metastasis-promoting factor in cancer progression. High HSF1 expression was associated with worse mCRPC prognosis, and H1KO attenuated metastatic growth of mCRPC cells. mCRPC cells showed distinct HSF1 transcriptome and cistrome profiles compared with CRPC cells. HSF1 protein and phosphorylation levels, genome-wide HSF1 occupancy and HSF1 target gene expression were greatly increased in mCRPC cells, suggesting that HSF1 is hyperactivated and drives a unique transcriptional program in metastatic cancer.

Accumulating evidence has demonstrated that DBC1 acts as a coregulator of various TFs in cancer cells^26^. Although a previous study showed that DBC1 has corepressor activity for HSF1^48^, our results suggest that DBC1 can act as both a coactivator and corepressor for HSF1 function. DBC1 cooperated with HSF1 to drive a transcriptional program critical for mCRPC progression, and the positive and negative target genes of HSF1 were downregulated and upregulated, respectively, by D1KO. Moreover, DBC1 was required for the chromatin occupancy of HSF1, the expression of SE-associated HSF1 target genes and the metastasis of mCRPC cells. For instance, DBC1 was required for HSF1 binding to and HSF1-directed SE formation on the MMP11 gene, and HSF1–DBC1 axis-induced MMP11 expression was also required for the metastatic properties of mCRPC cells. These results indicate a critical coregulator role of DBC1 in the HSF1-mediated transcription program promoting metastasis and that MMP11 is a key target gene of HSF1–DBC1 axis for promoting the metastatic potential of mCRPC cells (Fig. 6n).

HSF1 is subjected to diverse posttranslational modifications including phosphorylation and ubiquitination, which increase the complexity of HSF1 regulation^39^. DYRK2 phosphorylates, stabilizes and activates HSF1, thus promoting tumor growth by maintaining proteostasis in cancer cells^32^. Moreover, highly selective DYRK2 inhibitors effectively suppressed PCa tumor growth^49^, suggesting DYRK2 as a potential therapeutic target for PCa treatment. Notably, DBC1 enhanced DYRK2-mediated HSF1 phosphorylation and stabilization by increasing HSF1-DYRK2 interaction (Fig. 6n). In addition, we identified CHIP as a novel E3 ligase for HSF1 ubiquitination and degradation. DBC1 inhibited CHIP-mediated HSF1 ubiquitination by blocking the CHIP–HSF1 interaction (Fig. 6n). It should be noted that the finding of CHIP-mediated HSF1 ubiquitination and degradation was unexpected, as prior studies have shown that CHIP promotes HSF1 activity independently of its E3 ligase activity by facilitating HSF1 trimerization and nuclear localization^50,51^. The reasons for these conflicting results remain unknown. However, CHIP has dual functions as a cochaperone and ubiquitin E3 ligase. For example, CHIP acts as a chaperone for IRF-1, a TF involved in cancer progression, to promote IRF-1 stability in unstressed cells but functions as an E3 ligase to trigger ubiquitination-dependent degradation of IRF-1 under stress conditions^52^. In addition, in cooperation with HSP70/HSP90, CHIP ubiquitinates p53 for proteasomal degradation^53^. However, CHIP can act as a chaperone, independent of HSPs, to protect p53 from inactivation under stress conditions^53^. Thus, the role and mechanism of CHIP in stabilizing and degrading client proteins are likely to be very complex, and its dual-functional activity may be regulated in a cell type- and stress type-specific manner and in a binding partner-dependent manner.

HSF1 provides critical stress relief and confers metastatic and survival advantages to cancer cells exposed to myriad stresses, including genotoxic, proteotoxic, hypoxic and shear stresses, in TMEs and during metastasis. Thus, deciphering mechanisms underlying HSF1 stabilization and activation will provide useful information for developing novel therapeutic approaches for the treatment of a broad range of metastatic cancers and allow significant progress in our understanding of metastatic cancer biology. Here, we demonstrated that DBC1 functions as a key regulator of HSF1 stability and activity by enhancing HSF1 trimerization and phosphorylation and inhibiting HSF1 ubiquitination, thereby increasing the transcriptional activity and genome-wide binding of HSF1 (Fig. 6n). Importantly, high DBC1 expression was associated with mCRPC progression, and HSF1–DBC1 double-high expression was correlated with worse prognosis in mCRPC. Together with our previous finding showing a role of DBC1 in promoting CRPC by regulating AR/AR-V7 activity^22^, we propose DBC1 as a critical coregulator of two main pathways, AR/AR-V7 and HSF1 pathways, involved in PCa progression and metastasis. Thus, our findings provide a rationale for targeting DBC1 as a promising therapeutic approach to simultaneously block AR/AR-V7 and HSF1 pathways in mCRPC. In conclusion, our work demonstrates the critical role of HSF1 as a metastasis-promoting TF and a novel regulatory mechanism of HSF1 by DBC1. Our study highlights the important role of DBC1 in HSF1-mediated PCa progression and provides insights into regulatory mechanisms underlying HSF1 stabilization and activation in advanced and metastatic cancers. Thus, targeting the HSF1–DBC1 axis could be a promising therapeutic strategy for metastatic cancers.

## Supplementary information

Supplementary Information

## Data Availability

RNA-seq and ChIP-seq data files were deposited at NCBI GEO under SuperSeries accession codes GSE276301 and GSE276302, respectively (https://www.ncbi.nlm.nih.gov/geo). UCSC genome browser tracks of our processed ChIP-Seq data are available as a resource at https://genome.ucsc.edu/s/Sue%20Jin%20Moon/JeongKim.

## References

[1] Morimoto, R. I. The heat shock response: systems biology of proteotoxic stress in aging and disease. *Cold Spring Harb. Symp. Quant. Biol.* **76**, 91–99 (2011). 22371371 10.1101/sqb.2012.76.010637

[2] Kmiecik, S. W. & Mayer, M. P. Molecular mechanisms of heat shock factor 1 regulation. *Trends Biochem. Sci.* **47**, 218–234 (2022). 34810080 10.1016/j.tibs.2021.10.004

[3] Gomez-Pastor, R., Burchfiel, E. T. & Thiele, D. J. Regulation of heat shock transcription factors and their roles in physiology and disease. *Nat. Rev. Mol. Cell Biol.* **19**, 4–19 (2018). 28852220 10.1038/nrm.2017.73 PMC5794010

[4] Zou, J., Guo, Y., Guettouche, T., Smith, D. F. & Voellmy, R. Repression of heat shock transcription factor HSF1 activation by HSP90 (HSP90 complex) that forms a stress-sensitive complex with HSF1. *Cell* **94**, 471–480 (1998). 9727490 10.1016/s0092-8674(00)81588-3

[5] Chin, Y. et al. Targeting HSF1 for cancer treatment: mechanisms and inhibitor development. *Theranostics* **13**, 2281–2300 (2023). 37153737 10.7150/thno.82431 PMC10157728

[6] Puustinen, M. C. & Sistonen, L. Molecular mechanisms of heat shock factors in cancer. *Cells* **9**, 1202 (2020). 10.3390/cells9051202 PMC7290425 32408596

[7] Cyran, A. M. & Zhitkovich, A. Heat shock proteins and HSF1 in cancer. *Front. Oncol.* **12**, 860320 (2022). 35311075 10.3389/fonc.2022.860320 PMC8924369

[8] Mendillo, M. L. et al. HSF1 drives a transcriptional program distinct from heat shock to support highly malignant human cancers. *Cell* **150**, 549–562 (2012). 22863008 10.1016/j.cell.2012.06.031 PMC3438889

[9] Levi-Galibov, O. et al. Heat shock factor 1-dependent extracellular matrix remodeling mediates the transition from chronic intestinal inflammation to colon cancer. *Nat. Commun.* **11**, 6245 (2020). 33288768 10.1038/s41467-020-20054-x PMC7721883

[10] Dong, B. et al. Targeting therapy-resistant prostate cancer via a direct inhibitor of the human heat shock transcription factor 1. *Sci. Transl. Med.* **12**, eabb5647 (2020). 10.1126/scitranslmed.abb5647 PMC10571035 33328331

[11] Feng, Q. & He, B. Androgen receptor signaling in the development of castration-resistant prostate cancer. *Front. Oncol.* **9**, 858 (2019). 31552182 10.3389/fonc.2019.00858 PMC6738163

[12] Shafi, A. A., Yen, A. E. & Weigel, N. L. Androgen receptors in hormone-dependent and castration-resistant prostate cancer. *Pharm. Ther.* **140**, 223–238 (2013). 10.1016/j.pharmthera.2013.07.003 23859952

[13] Lu, J., Van der Steen, T. & Tindall, D. J. Are androgen receptor variants a substitute for the full-length receptor?. *Nat. Rev. Urol.* **12**, 137–144 (2015). 25666893 10.1038/nrurol.2015.13

[14] Kulasegaran, T. & Oliveira, N. Metastatic castration-resistant prostate cancer: advances in treatment and symptom management. *Curr. Treat. Options Oncol.* **25**, 914–931 (2024). 38913213 10.1007/s11864-024-01215-2 PMC11236885

[15] Carlin, B. I. & Andriole, G. L. The natural history, skeletal complications, and management of bone metastases in patients with prostate carcinoma. *Cancer* **88**, 2989–2994 (2000). 10898342 10.1002/1097-0142(20000615)88:12+<2989::aid-cncr14>3.3.co;2-h

[16] Azad, A. A., Zoubeidi, A., Gleave, M. E. & Chi, K. N. Targeting heat shock proteins in metastatic castration-resistant prostate cancer. *Nat. Rev. Urol.* **12**, 26–36 (2015). 25512207 10.1038/nrurol.2014.320

[17] Imamura, Y. & Sadar, M. D. Androgen receptor targeted therapies in castration-resistant prostate cancer: Bench to clinic. *Int J. Urol.* **23**, 654–665 (2016). 27302572 10.1111/iju.13137 PMC6680212

[18] Wyatt, A. W. & Gleave, M. E. Targeting the adaptive molecular landscape of castration-resistant prostate cancer. *EMBO Mol. Med.* **7**, 878–894 (2015). 25896606 10.15252/emmm.201303701 PMC4520654

[19] Yu, E. J. et al. Reciprocal roles of DBC1 and SIRT1 in regulating estrogen receptor alpha activity and co-activator synergy. *Nucleic Acids Res.* **39**, 6932–6943 (2011). 21596782 10.1093/nar/gkr347 PMC3167635

[20] Kim, H. J., Kim, S. H., Yu, E. J., Seo, W. Y. & Kim, J. H. A positive role of DBC1 in PEA3-mediated progression of estrogen receptor-negative breast cancer. *Oncogene* **34**, 4500–4508 (2015). 25417701 10.1038/onc.2014.381

[21] Fu, J. et al. Deleted in breast cancer 1, a novel androgen receptor (AR) coactivator that promotes AR DNA-binding activity. *J. Biol. Chem.* **284**, 6832–6840 (2009). 19126541 10.1074/jbc.M808988200 PMC2652261

[22] Moon, S. J. et al. DBC1 promotes castration-resistant prostate cancer by positively regulating DNA binding and stability of AR-V7. *Oncogene* **37**, 1326–1339 (2018). 29249800 10.1038/s41388-017-0047-5

[23] Yu, E. J. et al. Positive regulation of β-catenin–PROX1 signaling axis by DBC1 in colon cancer progression. *Oncogene* **35**, 3410–3418 (2016). 26477307 10.1038/onc.2015.401 PMC5058359

[24] Kim, H. J., Moon, S. J., Hong, S., Won, H. H. & Kim, J. H. DBC1 is a key positive regulator of enhancer epigenomic writers KMT2D and p300. *Nucleic Acids Res.* **50**, 7873–7888 (2022). 35801925 10.1093/nar/gkac585 PMC9371912

[25] Kim, J. E., Chen, J. & Lou, Z. DBC1 is a negative regulator of SIRT1. *Nature* **451**, 583–586 (2008). 18235501 10.1038/nature06500

[26] Kim, H. J., Moon, S. J. & Kim, J. H. Mechanistic insights into the dual role of CCAR2/DBC1 in cancer. *Exp. Mol. Med.* **55**, 1691–1701 (2023). 37524873 10.1038/s12276-023-01058-1 PMC10474295

[27] Li, Z. et al. Inhibition of SUV39H1 methyltransferase activity by DBC1. *J. Biol. Chem.* **284**, 10361–10366 (2009). 19218236 10.1074/jbc.M900956200 PMC2667723

[28] Chini, C. C., Escande, C., Nin, V. & Chini, E. N. HDAC3 is negatively regulated by the nuclear protein DBC1. *J. Biol. Chem.* **285**, 40830–40837 (2010). 21030595 10.1074/jbc.M110.153270 PMC3003384

[29] Qin, B. et al. DBC1 functions as a tumor suppressor by regulating p53 stability. *Cell Rep.* **10**, 1324–1334 (2015). 25732823 10.1016/j.celrep.2015.01.066 PMC4351187

[30] Moon, S. J. et al. Bruceantin targets HSP90 to overcome resistance to hormone therapy in castration-resistant prostate cancer. *Theranostics* **11**, 958–973 (2021). 33391515 10.7150/thno.51478 PMC7738850

[31] Crnalic, S., Lofvenberg, R., Bergh, A., Widmark, A. & Hildingsson, C. Predicting survival for surgery of metastatic spinal cord compression in prostate cancer: a new score. *Spine* **37**, 2168–2176 (2012). 22648028 10.1097/BRS.0b013e31826011bc

[32] Moreno, R. et al. The stress-responsive kinase DYRK2 activates heat shock factor 1 promoting resistance to proteotoxic stress. *Cell Death Differ.* **28**, 1563–1578 (2021). 33268814 10.1038/s41418-020-00686-8 PMC8166837

[33] Jacobs, C. et al. HSF1 inhibits antitumor immune activity in breast cancer by suppressing CCL5 to block CD8^+^ T-cell recruitment. *Cancer Res.* **84**, 276–290 (2024). 37890164 10.1158/0008-5472.CAN-23-0902 PMC10790131

[34] Dastidar, S. G. et al. Transcriptional responses of cancer cells to heat shock-inducing stimuli involve amplification of robust HSF1 binding. *Nat. Commun.* **14**, 7420 (2023). 37973875 10.1038/s41467-023-43157-7 PMC10654513

[35] Himanen, S. V., Puustinen, M. C., Da Silva, A. J., Vihervaara, A. & Sistonen, L. HSFs drive transcription of distinct genes and enhancers during oxidative stress and heat shock. *Nucleic Acids Res.* **50**, 6102–6115 (2022). 35687139 10.1093/nar/gkac493 PMC9226494

[36] Welti, J. et al. NXP800 activates the unfolded protein response, altering AR and E2F function to impact castration-resistant prostate cancer growth. *Clin. Cancer Res.* **31**, 1109–1126 (2025). 39787247 10.1158/1078-0432.CCR-24-2386 PMC11911806

[37] Gorodetska, I. et al. Blood-based detection of MMP11 as a marker of prostate cancer progression regulated by the ALDH1A1-TGF-beta1 signaling mechanism. *J. Exp. Clin. Cancer Res.* **44**, 105 (2025). 40122809 10.1186/s13046-025-03299-6 PMC11931756

[38] Tan, B. et al. MMP11 and MMP14 contribute to the interaction between castration-resistant prostate cancer and adipocytes. *Am. J. Cancer Res.* **13**, 5934–5949 (2023). 38187060 PMC10767328

[39] Fujimoto, M., Takii, R. & Nakai, A. Regulation of HSF1 transcriptional complexes under proteotoxic stress: Mechanisms of heat shock gene transcription involve the stress-induced HSF1 complex formation, changes in chromatin states, and formation of phase-separated condensates: mechanisms of heat shock gene transcription involve the stress-induced HSF1 complex formation, changes in chromatin states, and formation of phase-separated condensates. *Bioessays* **45**, e2300036 (2023). 37092382 10.1002/bies.202300036

[40] Kourtis, N. et al. FBXW7 modulates cellular stress response and metastatic potential through HSF1 post-translational modification. *Nat. Cell Biol.* **17**, 322–332 (2015). 25720964 10.1038/ncb3121 PMC4401662

[41] Kim, E. et al. NEDD4-mediated HSF1 degradation underlies α-synucleinopathy. *Hum. Mol. Genet.* **25**, 211–222 (2016). 26503960 10.1093/hmg/ddv445 PMC4706110

[42] Kumar, S., Basu, M. & Ghosh, M. K. Chaperone-assisted E3 ligase CHIP: a double agent in cancer. *Genes Dis.* **9**, 1521–1555 (2022). 36157498 10.1016/j.gendis.2021.08.003 PMC9485218

[43] Liu, C. et al. Proteostasis by STUB1/HSP70 complex controls sensitivity to androgen receptor targeted therapy in advanced prostate cancer. *Nat. Commun.* **9**, 4700 (2018). 30446660 10.1038/s41467-018-07178-x PMC6240084

[44] Biswas, K., Sarkar, S., Said, N., Brautigan, D. L. & Larner, J. M. Aurora B kinase promotes CHIP-dependent degradation of HIF1α in prostate cancer cells. *Mol. Cancer Ther.* **19**, 1008–1017 (2020). 31848297 10.1158/1535-7163.MCT-19-0777

[45] Takahashi, S. et al. Aneusomies of chromosomes 8 and Y detected by fluorescence in situ hybridization are prognostic markers for pathological stage C (pt3N0M0) prostate carcinoma. *Clin. Cancer Res* **2**, 137–145 (1996). 9816100

[46] Jenkins, R. B., Qian, J., Lieber, M. M. & Bostwick, D. G. Detection of c-Myc oncogene amplification and chromosomal anomalies in metastatic prostatic carcinoma by fluorescence in situ hybridization. *Cancer Res.* **57**, 524–531 (1997). 9012485

[47] Kourtis, N. et al. Oncogenic hijacking of the stress response machinery in T cell acute lymphoblastic leukemia. *Nat. Med.* **24**, 1157–1166 (2018). 30038221 10.1038/s41591-018-0105-8 PMC6082694

[48] Raynes, R. et al. The SIRT1 modulators AROS and DBC1 regulate HSF1 activity and the heat shock response. *PLoS One* **8**, e54364 (2013). 23349863 10.1371/journal.pone.0054364 PMC3548779

[49] Yuan, K. et al. Targeting dual-specificity tyrosine phosphorylation-regulated kinase 2 with a highly selective inhibitor for the treatment of prostate cancer. *Nat. Commun.* **13**, 2903 (2022). 35614066 10.1038/s41467-022-30581-4 PMC9133015

[50] Dai, Q. et al. CHIP activates HSF1 and confers protection against apoptosis and cellular stress. *EMBO J.* **22**, 5446–5458 (2003). 14532117 10.1093/emboj/cdg529 PMC213783

[51] Huang, C. Y. et al. Doxorubicin attenuates CHIP-guarded HSF1 nuclear translocation and protein stability to trigger IGF-IIR-dependent cardiomyocyte death. *Cell Death Dis.* **7**, e2455 (2016). 27809308 10.1038/cddis.2016.356 PMC5260882

[52] Narayan, V., Pion, E., Landre, V., Muller, P. & Ball, K. L. Docking-dependent ubiquitination of the interferon regulatory factor-1 tumor suppressor protein by the ubiquitin ligase CHIP. *J. Biol. Chem.* **286**, 607–619 (2011). 20947504 10.1074/jbc.M110.153122 PMC3013021

[53] Esser, C., Scheffner, M. & Hohfeld, J. The chaperone-associated ubiquitin ligase CHIP is able to target p53 for proteasomal degradation. *J. Biol. Chem.* **280**, 27443–27448 (2005). 15911628 10.1074/jbc.M501574200

