# Supplementary material for: HSF1–DBC1 axis drives prostate cancer progression by activating a metastatic transcriptional program
Source: Exp Mol Med. 2025 Oct 1;57(10):2277–91. doi: 10.1038/s12276-025-01545-7 (PMC12586603; doi:10.1038/s12276-025-01545-7)
Supplement: Supplementary file 1 — Supplementary Information [file 12276_2025_1545_MOESM1_ESM.pdf]

SUPPLEMENTARY INFORMATION

SUPPLEMENTARY MATERIALS AND METHODS

Cell culture and transient transfection

All cell lines used were obtained from American Type Culture Collection (ATCC) or Korean Cell Line Bank (KCLB) and regularly tested for mycoplasma contamination and authenticity using STR genotyping. 22RV1, 22RV1-H1KO, 22RV1-D1KO, SM1, SM1-H1KO, SM1-D1KO, KM1, and LM1 were cultured in RPMI 1640 with 10% fetal bovine serum (FBS). 293T and PC3 cells were grown in DMEM with 10% FBS. For transient transfection, cells were transfected with expression plasmids and/or HSF1-regulated LUC reporters and control reporter pRL-SV40 (Promega) using Lipofectamine 3000 (Invitrogen) or jetPEI (Polyplus). For proteotoxic stress, cells were incubated at 42°C for the indicated time points in figure legends. Each experiment was repeated independently at least three times.

Generation of metastatic CRPC cell lines

To generate cell lines with enhanced metastatic propensity, $3x10^6$ 22RV1-LUC cells (expressing luciferase and hygromycin resistance genes) suspended in 100 μl Matrigel/PBS (50:50 mixture) were subcutaneously injected into 6-week-old male athymic BALB/c nu/nu mice (Orient Bio). After 3 weeks, primary tumors were harvested, washed, minced, and further digested with Liberase TM (Sigma-Adrich) as described previously[1]. Tumor 22RV1-LUC cells (22RV1-LUC-T1) were enriched by passaging 5 times in medium containing hygromycin (200 μg/ml) to remove mouse cell contamination. 22RV1-LUC-T1 cells were injected into the left cardiac ventricle of male nude mice under ultrasound guidance (VisualSonics Vevo 2100 imaging system). Bioluminescence imaging was performed weekly to monitor metastasis formation using the IVIS Spectrum Imaging System (Xenogen, PerkinElmer). 22RV1-LUC-T1 cells that metastasized to spinal cord (SM1), kidney (KM1), and liver (LM1) were isolated and enriched by subculturing in medium containing hygromycin.

Plasmids

HSF1 was PCR-amplified from 22RV1 cDNA and cloned into pSG5.HA, pcDNA3.1-3xFLAG, and pETDuet-1 (Novagen). PCR-amplified DYRK2 from 22RV1 cDNA was cloned into pSG5.HA and p3XFLAG-CMV-10 (Sigma-Aldrich). The human HSP90β expression vector was obtained from Addgene (HSP90 HA, #22487). HSF1 S320D/S326D mutant and HSP90β E42A mutant were generated with the QuikChange site-directed mutagenesis kit (Agilent Technologies). MMP11 was PCR-amplified from

SW480 cDNA and cloned into pSG5.HA. To generate CRISPR/Cas9 vectors, single guide RNA (sgRNA) oligonucleotides targeting human HSF1 (sgHSF1#1) or DBC1 (sgDBC1#1 and #3) were annealed and cloned into the BbsI site of pSpCas9(BB)-2A-GFP (PX458) (Addgene, #48138). The sgRNA sequences are listed in Supplementary Table 1. The 6xHSE sequence was PCR-amplified from pMlucF 6xHSE, which was kindly provided by Thomas Czerny (University of Applied Science, Austria), and cloned into pTA-LUC (Clontech). The HSE regions of HSPA1L (nucleotides -290 to +603 relative to transcription start site) and NEAT1 (nucleotides -506 to -379 relative to the transcription start site) were amplified from 22RV1 genomic DNA and cloned into pGL3-Basic (Promega) and pTA-LUC, respectively. PCR-amplified 6xHSE-TATA (from p6xHSE-TA-LUC), NEAT1-TATA (from pNEAT1-TA-LUC), and HSPA1L promoter (from pGL3-HSPA1L-LUC) were cloned into pNL1.3[secNluc] (Promega). Lentiviral vectors expressing shRNAs were generated by inserting annealed oligonucleotides encoding shRNAs specific for HSF1, DYRK2, or MMP11 into pLKO.1 (Sigma-Aldrich). The oligonucleotides used for shRNA constructs are listed in Supplementary Table 1. PCR-amplified 3xFLAG-DBC1 was cloned into lentiviral vector pHR.CMV.FLAG.IRES-Hygro[2]. Lentiviral particles were produced as described previously [3]. The following plasmids were described previously: pSG5.HA-DBC1, pcDNA3.1-DBC1-V5/His, p3×FLAG.CMV10-DBC1, pGEX-4T-1-DBC1, pcDNA3.1-CHIP-V5/His, pLKO.1-shDBC1#5, and pHR.CMV.puro.Sin8-shNS[2-6].

Antibodies

The following antibodies were used in this study: anti-HSF1, ADI-SPA-901-F (Enzo Biochem Inc.) and #4356 (Cell Signaling Technology); anti-HSF1 phospho-S320, ab76183 (Abcam); anti-HSF1 phospho-S326, ab115702 (Abcam) and CSB-RA010791A326phHU (Cusabio Technology); anti-DBC1, A300-434A (Bethyl Laboratories) and A7126 (ABclonal); anti-H3K27ac, ab4729 (Abcam); anti-DYRK2, #11921 (Cell Signaling Technology) and A24467 (ABclonal); anti-MMP11, CSB-PA003254 (Cusabio Technology); anti-CHIP, #2080 (Cell Signaling Technology); anti-Ubiquitin, sc-8017 (Santa Cruz Biotechnology); anti-GAPDH, AC001 (ABclonal); anti-HA, 3F10 (Roche); anti-HA-agarose, A2095 (Sigma-Aldrich); anti-FLAG M2 and M2-agarose (Sigma-Aldrich); anti-V5, #R960-25 (Invitrogen); anti-His, sc-8036 (Santa Cruz Biotechnology).

Cell proliferation, colony formation, migration, invasion, and sphere formation assays

Cell proliferation, colony formation, migration, invasion, and sphere formation assays were performed as previously described[1,4,6,7]. Briefly, cell proliferation was determined by MTT assays (Promega). In some MTT assays, cells were treated with enzalutamide (BioVision Technologies) or docetaxel (SelleckChem), and IC50 values were determined by non-linear regression using PRISM v5.0 (GraphPad). For colony

formation assays, cells were plated at a density of $1x10^3$ cells/well in 6-well plates, and colonies were stained with crystal violet. Stained colonies were solubilized in 10% SDS, and absorbance was measured at 570 nm. Two-chamber migration and invasion assays were performed in 24-well plates at $5x10^4$ cells/well using Transwell inserts (Costar) coated with fibronectin (Sigma-Aldrich) and Matrigel (BD Biosciences)/fibronectin, respectively. For sphere formation assays, cells were cultured in suspension at $2x10^2$ cells/well in 96-well ultralow attachment plates (Costar) with serum-free DMEM-F12 (1:1) supplemented with 20 ng/μl hEGF, 10 ng/μl bFGF, 1x B27, and 1x N-2 (Life Technologies). Numbers of spheres with a diameter of >100 μm or less were counted between days 10 and 14.

Xenograft experiments

Animal experiments were conducted in accordance with the principles of the Declaration of Helsinki principles and with the approval of the Institutional Animal Care and Use Committee of Laboratory Animal Research Center at Samsung Medical Center, ensuring the highest standards of animal welfare and ethical conduct throughout the research process. The maximum tumor size/burden (≤ 1,500 mm^3) was not exceeded in our mouse experiments. Mouse xenograft experiments were performed as described above and in previous studies[1,4,6,7]. Briefly, $3x10^6$ 22RV1-LUC or 22RV1-H1KO-LUC cells were injected subcutaneously into the left flank of 6-week-old male athymic BALB/c nu/nu mice (n=5, each group) (Orient Bio, Korea). Tumor volumes were measured every two days, and in vivo bioluminescence was measured at day 21. For metastasis experiments, $2x10^5$ of SM1, SM1-H1KO, and SM1-D1KO cells were injected into the left cardiac ventricle of 6-week-old male nude mice (n=10, each group) guided by ultrasound imaging (VisualSonics Vevo 2100 imaging system). Bioluminescence imaging was performed once a week to monitor tumor metastasis using the IVIS Spectrum Imaging System (Xenogen, PerkinElmer).

RNA-seq analysis

RNA-seq analysis was performed as previously described[1,5]. Briefly, total RNAs were isolated using RNeasy Plus Kit (Qiagen). RNA-seq libraries were prepared using the TruSeq Stranded mRNA Library Prep Kit (Illumina) and sequenced on a NovaSeq 6000 (Illumina) platform in paired-end, 101 bp mode. Trimmed reads were mapped to the human reference genome (GRCh37/hg19). Gene expression levels were quantified as fragments per kilobase of transcript per million mapped reads (FPKM), and differentially expressed genes were determined using DESeq2 with the cut-off of log2FC ≥ 0.58 (fold change ≥ 1.5) and P < 0.05. Volcano plot, heatmap, scatter plot, and Pearson correlation analyses between RNA-seq datasets were performed using R software package (v4.2.1).

ChIP-seq analysis

ChIP assays were performed as described previously using a SimpleChIP Plus Enzymatic Chromatin IP kit (Cell Signaling Technology) following the manufacturer's instructions[5]. ChIP-seq libraries were constructed using the TruSeq ChIP Library Preparation Kit (Illumina) and sequenced using a NovaSeq 6000 (Illumina) to yield 70–100 million 101 bp paired-end reads per sample. Raw sequence reads were trimmed with Trimmomatic v0.38 and aligned to the human reference genome (assembly hg19) using Bowtie v1.1.2. ChIP-seq peak calling was performed with MACS2 v2.1.1 using respective chromatin input as a background control. Control HSF1 ChIP-seq and DBC1 ChIP-seq were also performed in H1KO and D1KO cells, respectively, and ChIP-seq peaks detected in KO cells, defined as background signals, were subtracted from ChIP-seq data sets in wild-type cells to remove non-specific peaks. ChIPpeakAnno v3.36.1 was used to determine overlapping peaks. MAnorm was used for quantitative comparison of ChIP-seq peaks ($P < 0.05$, $FC > 1.5$). ChIPseeker v1.16.1 was used to annotate peaks to the nearest genes. Heatmaps of ChIP-seq signals were generated using deepTools v3.5.4 on the Galaxy server (https://usegalaxy.eu/). ChIP-seq tracks were visualized in the IGV genome browser (https://igv.org/) using Bigwig files of ChIP-seq data. Motif enrichment analysis was performed using MEME-ChIP v5.5.5. Super-enhancers (SEs) were identified based on H3K27ac ChIP-seq signals using the Rank Ordering of SE (ROSE) algorithm with default parameters[8].

Real-time quantitative reverse transcription-PCR (qRT-PCR)

Total RNA was extracted using TRIzol reagent (Invitrogen), and qRT-PCR was performed using the One-step PrimeScript RT-PCR Kit (Takara Bio). qRT-PCR was performed in triplicate using gene-specific primers on QuantStudio 6 Flex real-time PCR system (Applied Biosystems). Data were normalized to GAPDH or β-actin mRNA levels. The primers used for qRT-PCR are listed in Supplementary Table 1.

Protein-protein interaction assays

Coimmunoprecipitation (CoIP) and GST pull-down assays were performed as described previously[5]. Briefly, for CoIP assays, cells were lysed in FLAG lysis buffer[5] containing phosphatase and protease inhibitor cocktails (Roche Diagnostics) and immunoprecipitated with control IgG or specific antibodies and Protein G Dynabeads (Invitrogen). For CoIP between HSF1 and HSP90β, the client-trapping mutant of HSP90β (HSP90β E42A) was used to strengthen the transient interaction between HSF1 and HSP90β. HSP90β E42A mutant binds ATP but is defective for ATP hydrolysis and trapped in the closed dimer conformation[9]. For GST pull-down assays, bacterially expressed, purified GST fusion proteins were incubated with in vitro-translated proteins in NETN buffer[5]. Bound proteins were analyzed by SDS-PAGE and immunoblot. In some experiments, SM1 cell lysates and immunopurified HSF1 were treated with

lambda protein phosphatase (NEB), following manufacturer's instructions, and used for CoIP and GST pull-down assays.

In vitro HSF1 cross-linking analysis

293T cell lysates transfected with expression vectors for HA-HSF1 and DBC1-V5 were cross-linked with 2 mM ethylene glycol bis(succinimidyl succinate) (EGS, Sigma-Aldrich) for 30 min and quenched with 75 mM glycine for 10 min. The cross-linking reactions were then analyzed by SDS-PAGE and immunoblot analysis with HA antibodies.

Ubiquitination assays

Ubiquitination assays were performed as described previously[1,4]. SM1 cells were transfected with expression plasmids for CHIP and DBC1 as indicated in figure legends or treated with a DYRK inhibitor. After 48 h transfection or 24 h inhibitor treatment, cells were treated with 10 μM MG132 for 4-5 h and then lysed in FLAG lysis buffer. HSF1 was immunoprecipitated, and its ubiquitination level was determined by immunoblot using anti-ubiquitin antibodies.

SUPPLEMENTARY FIGURES AND FIGURE LEGENDS

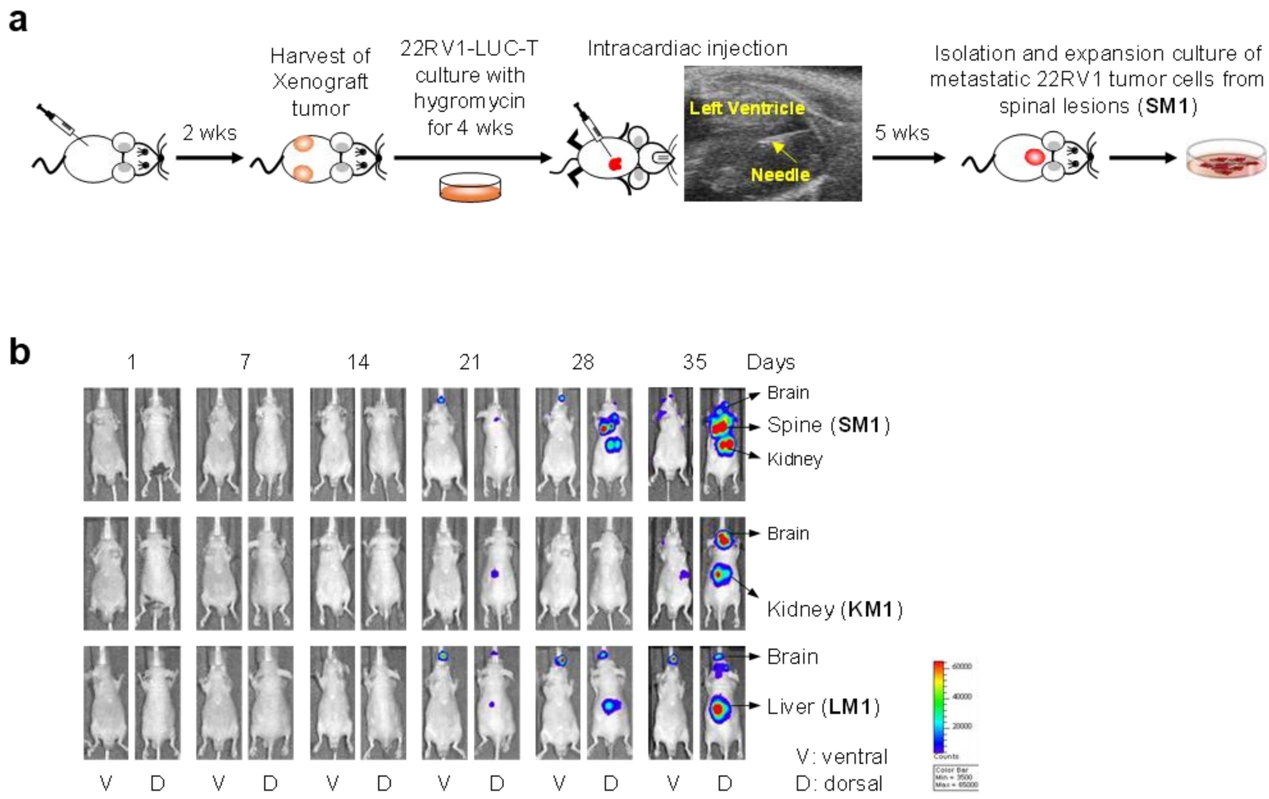

Supplementary Fig. 1. Establishment of metastatic 22RV1 derivative cell lines.

a Schematic diagram of establishment of metastatic 22RV1 derivative cell lines. i) 22RV1-LUC cells were subcutaneously injected into nude mice. ii) After 2 weeks, subcutaneous 22RV1 xenograft tumors were harvested, minced, and cultured (22RV1-LUC-T1). iii) 22RV1-LUC-T1 cells were intracardiacally injected into 6-week-old male athymic BALB/c nu/nu mice guided by ultrasound. iv) Metastatic tumors were monitored by bioluminescence imaging and harvested, and the cells from spine metastatic tumor were cultured and established as a metastatic 22RV1 cell line (SM1). **b** Representative bioluminescence images of the mice (dorsal and ventral sides) at the indicated days after intracardiac injection of 22RV1-LUC-T cells. In addition to SM1, cells from kidney and liver metastatic tumors were cultured and established as metastatic 22RV1 cell lines (KM1 and LM1).

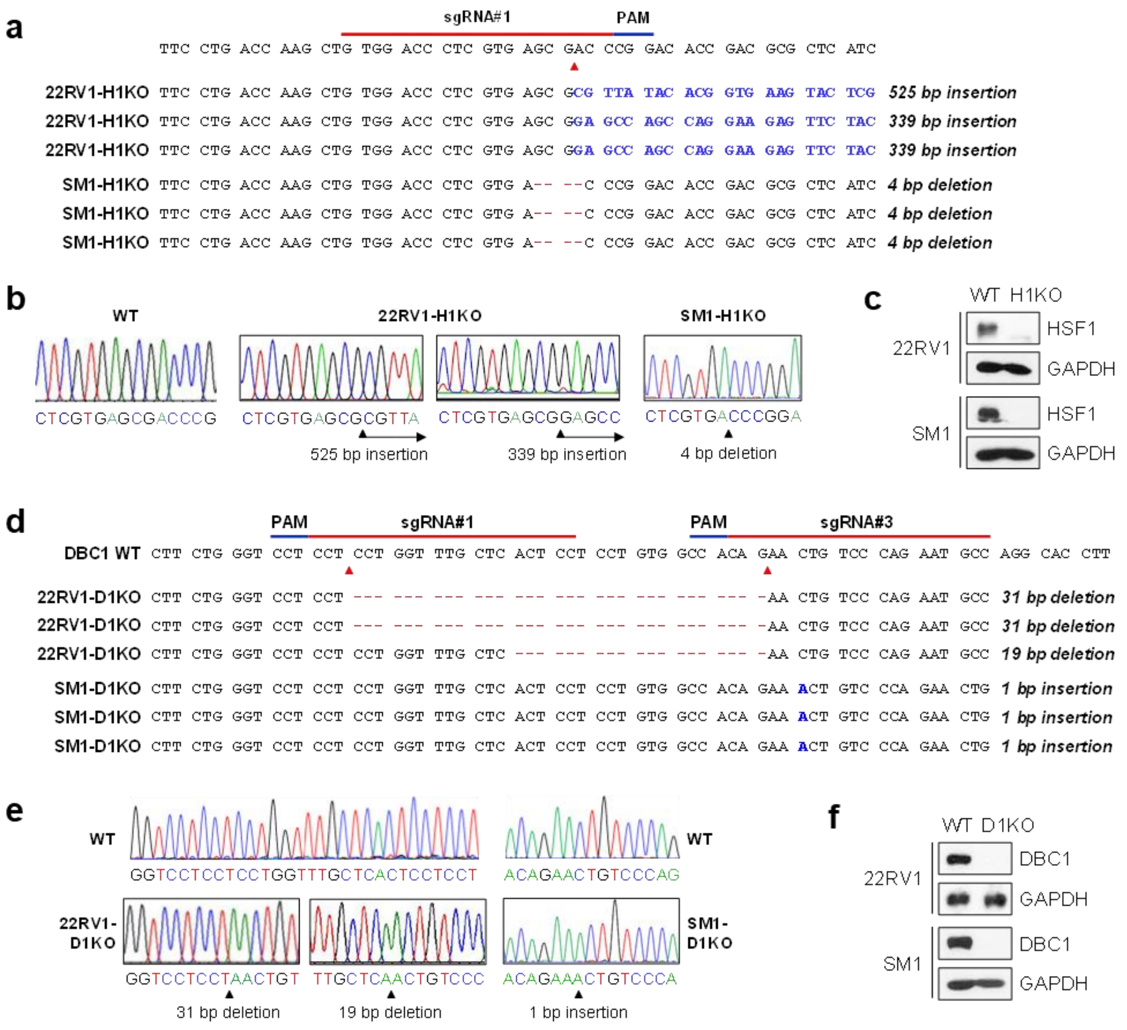

Supplementary Fig. 2. CRISPR/Cas9-mediated knock-out of HSF1 and DBC1 in 22RV1 and SM1 cells.

a-c A single guide RNA (sgRNA) was used to induce DNA double strand break at exon 1 of the HSF1 locus (a). The sgRNA and proto-spacer adjacent motif (PAM) are labeled with red and blue lines, respectively. Cas9 cleavage sites were indicated by red arrowhead. DNA sequencing of PCR products from the genomic DNA of the HSF1 KO (H1KO) cells revealed indel-mediated frameshift mutations at the target site. Sanger sequencing chromatograms (b) and immunoblots of HSF1 protein levels (c) in 22RV1, H1KO, SM1, and SM1-H1KO are shown. **d-f** Two sgRNAs used to target exon 3 of the DBC1 locus are labeled with red lines, and PAM is highlighted by blue lines (d). Red arrowheads indicate Cas9 cleavage sites. DNA sequencing of PCR products amplified from sgRNA-targeted sites showed indel-mediated frameshift mutations at the target sites. 22RV1 and SM1 cells carry three copies of chromosome 8 containing the HSF1 and DBC1 genes. Sanger sequencing chromatograms (e) and immunoblots of DBC1 protein levels (f) in 22RV1, D1KO, SM1, and SM1-D1KO are shown.

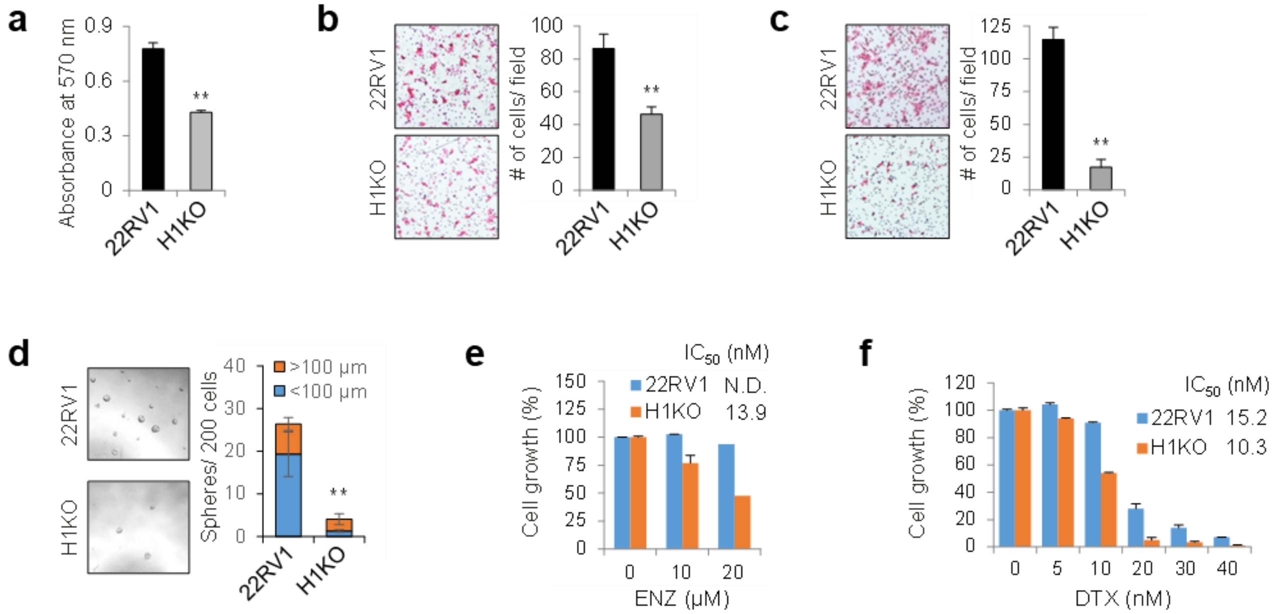

Supplementary Fig. 3. HSF1 is required for the metastatic potential of CRPC cells.
a-c Effects of HSF1 KO (H1KO) on cell proliferation (a), migration (b), and invasion (c) of 22RV1 cells. **P < 0.001. **d** Sphere formation analysis of 22RV1 and H1KO cells. **P<0.001. **e-f** 22RV1 and H1KO cells were treated with indicated concentrations of enzalutamide (ENZ) (e) and docetaxel (DTX) (f). Cell proliferation was detected by MTT assays, and IC50 values were determined.

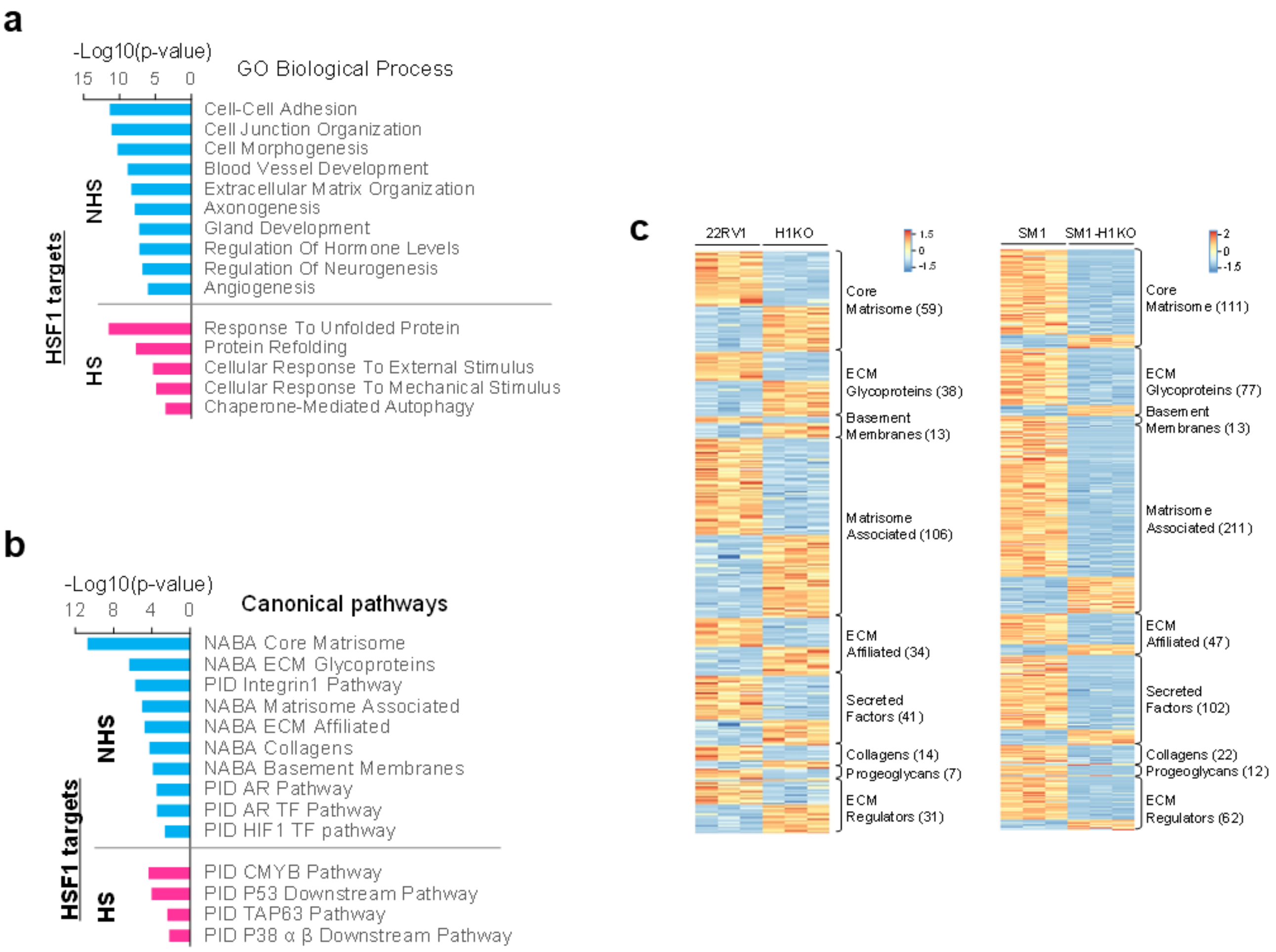

Supplementary Fig. 4. Hyperactivation and transcriptional reprogramming of HSF1 in mCRPC cells.

a-b Bar plots show enriched gene ontology (GO) biological processes (a) and canonical pathways (b) for HSF1 target genes in 22RV1 cells treated with or without HS. **c** Heatmaps showing differential matrisome gene expression between 22RV1 and H1KO (left) and between SM1 and SM1-H1KO (right) cells.

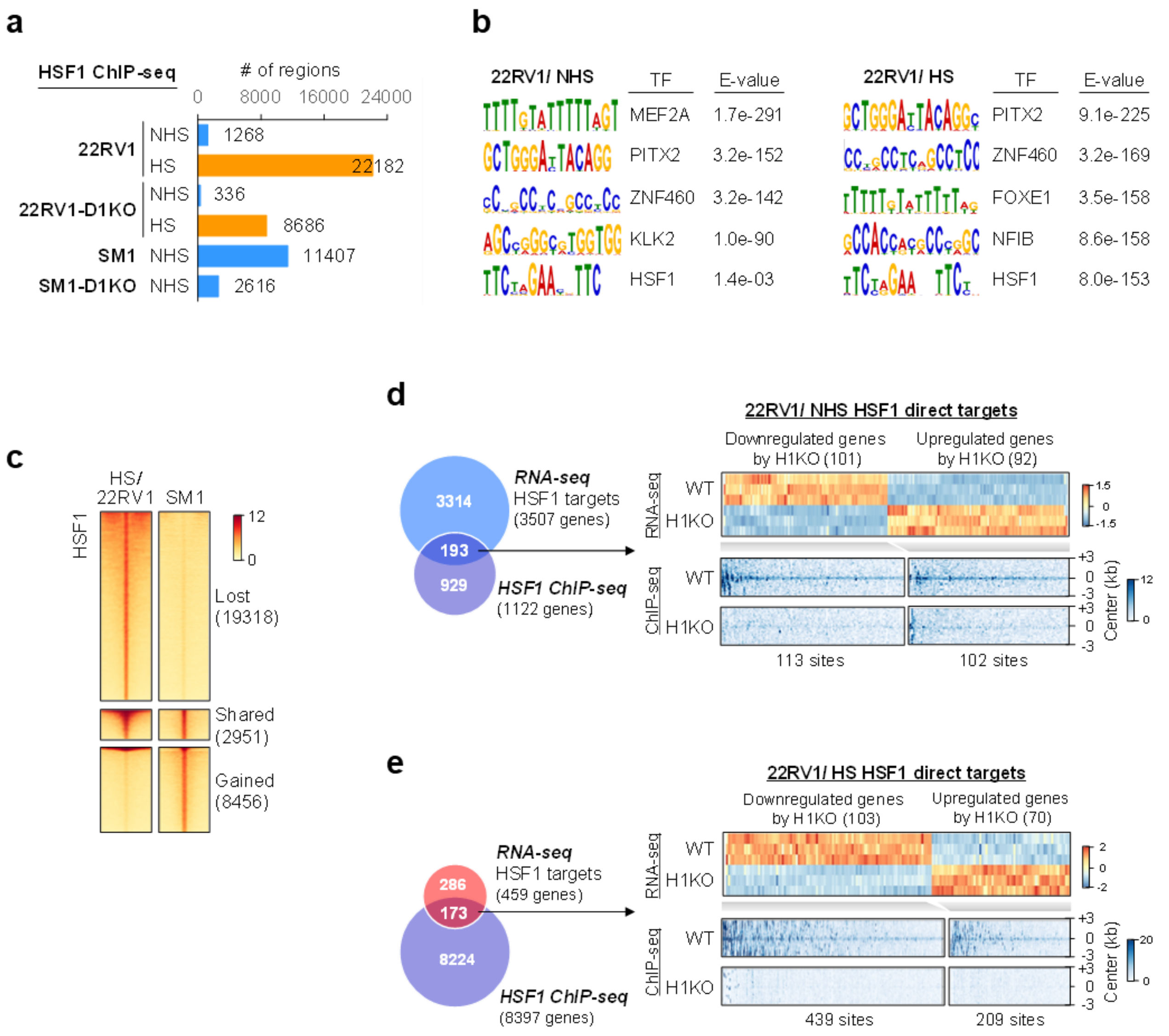

Supplementary Fig. 5. Genome-wide occupancy and direct targets of HSF1 in CRPC and mCRPC cells.

a Numbers of HSF1-bound chromatin regions in 22RV1 (NHS and HS), D1KO (NHS and HS), SM1 (NHS), and SM1-D1KO (NHS) cells. **b** MEME-ChIP motif analysis reveals the top enriched motifs in HSF1 binding sites in 22RV1 cells (NHS and HS). **c** Heatmaps of HSF1 ChIP-seq signals in 22RV1 (HS) versus SM1 (NHS) cells. **d-e** Venn diagrams showing the overlap of HSF1-regulated genes (RNA-seq) and HSF1-bound genes (ChIP-seq) in 22RV1 cells under NHS (d) and HS (e) conditions. Heatmaps showing the pattern of HSF1 direct target gene expression and HSF1 ChIP-seq signals in 22RV1 versus H1KO cells.

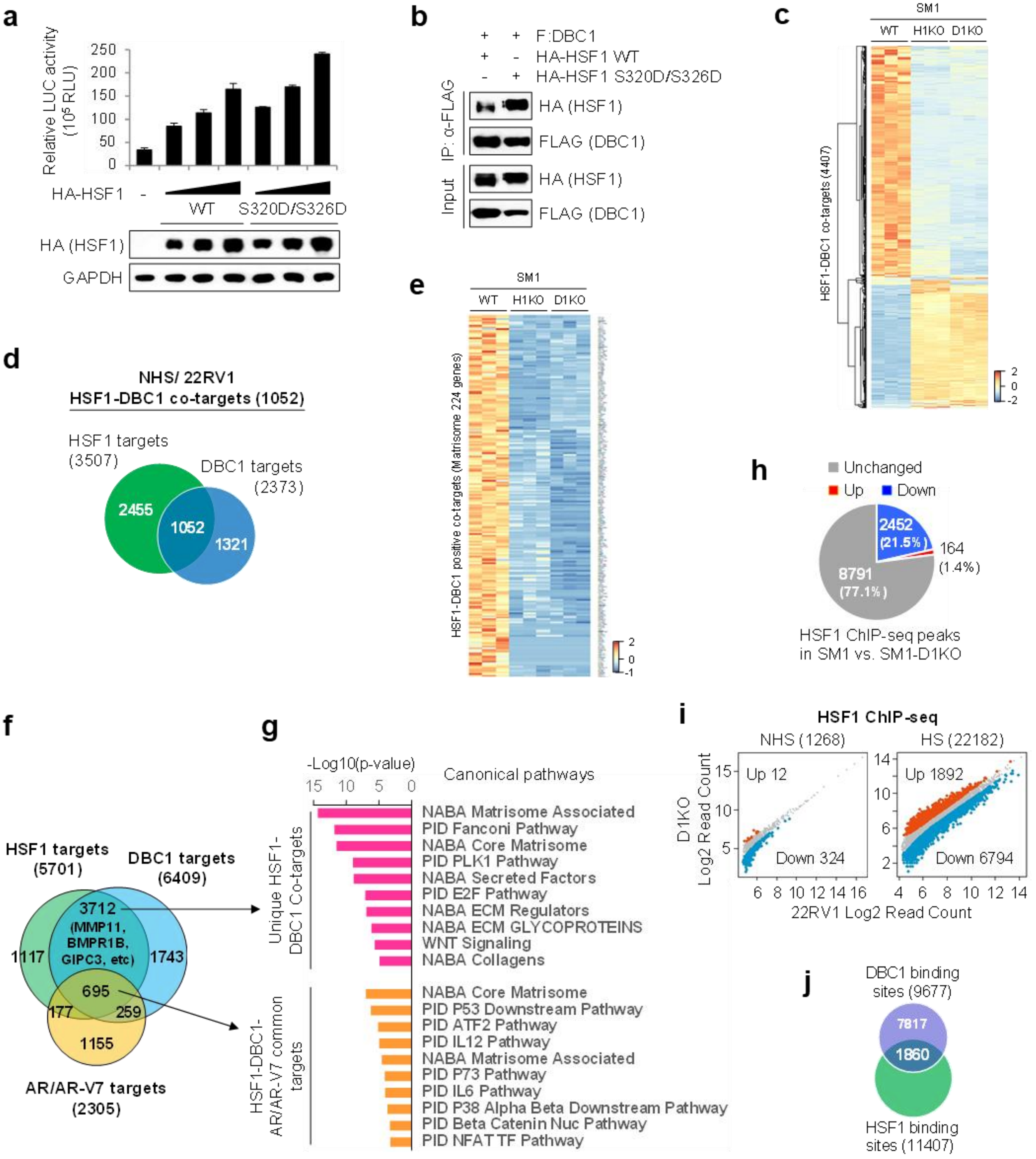

a
Relative LUC activity (10^5 RLU)
250
200
150
100
50
0
HA-HSF1
-
WT
S320D/S326D
HA (HSF1)
GAPDH
b
+ + F:DBC1
+ - HA-HSF1 WT
- + HA-HSF1 S320D/S326D
IP: α-FLAG
Input
HA (HSF1)
FLAG (DBC1)
HA (HSF1)
FLAG (DBC1)
c
SM1
WT H1KO D1KO
HSF1-DBC1 co-targets (4407)
2
0
-2
d
NHS/ 22RV1
HSF1-DBC1 co-targets (1052)
HSF1 targets (3507)
DBC1 targets (2373)
2455
1052
1321
e
SM1
WT H1KO D1KO
HSF1-DBC1 positive co-targets (Matrisome 224 genes)
2
0
-1
h
Unchanged
Up
Down
2452 (21.5%)
164 (1.4%)
8791 (77.1%)
HSF1 ChIP-seq peaks in SM1 vs. SM1-D1KO
f
HSF1 targets (5701)
DBC1 targets (6409)
3712 (MMP11, BMPR1B, GIPC3, etc)
1117
1743
695
177
259
1155
AR/AR-V7 targets (2305)
g
-Log10(p-value)
15 10 5 0
Canonical pathways
Unique HSF1-DBC1 Co-targets
NABA Matrisome Associated
PID Fanconi Pathway
NABA Core Matrisome
PID PLK1 Pathway
NABA Secreted Factors
PID E2F Pathway
NABA ECM Regulators
NABA ECM GLYCOPROTEINS
WNT Signaling
NABA Collagens
HSF1-DBC1-AR/AR-V7 common targets
NABA Core Matrisome
PID P53 Downstream Pathway
PID ATF2 Pathway
PID IL12 Pathway
NABA Matrisome Associated
PID P73 Pathway
PID IL6 Pathway
PID P38 Alpha Beta Downstream Pathway
PID Beta Catenin Nuc Pathway
PID NFAT TF Pathway
i
HSF1 ChIP-seq
NHS (1268)
HS (22182)
D1KO Log2 Read Count
Up 12
Down 324
Up 1892
Down 6794
22RV1 Log2 Read Count
j
DBC1 binding sites (9677)
7817
1860
HSF1 binding sites (11407)

Supplementary Fig. 6. DBC1 acts as a coregulator of HSF1 and is required for efficient chromatin binding of HSF1.

a Effects of phospho-mimetic mutation on HSF1 transcriptional activity. 293T cells were transfected with 6xHSE-LUC (pMlucF 6HSE) reporter and increasing amounts of pSG5.HA-HSF1 or pSG5.HA-HSF1 S320D/S326D, and luciferase assays were performed. Data are means ± s.d. (n = 3). Similar expression levels of transfected HSF1 wild-type (WT) and S320D/S326D mutant were ascertained by immunoblot with anti-HA antibody. **b** Effects of phospho-mimetic mutation on HSF1 binding to DBC1. 293T cells were transfected with the indicated constructs, and cell extracts were immunoprecipitated with FLAG-M2 agarose beads and analyzed by immunoblot with the indicated antibodies. **c** Heatmaps showing changes in HSF1-DBC1 co-target gene expression in SM1 versus SM1-H1KO or SM1-D1KO cells. **d** Venn diagram showing the overlap between HSF1 and DBC1 target genes in 22RV1 cells. **e** Heatmap showing changes in expression of matrisome genes among HSF1-DBC1 positive co-targets in SM1 versus SM1-H1KO or SM1-D1KO cells. **f** Venn diagram displaying the overlap between target genes for HSF1, DBC1, and AR/AR-V7 in SM1 cells. AR/AR-V7 target genes were identified by combinatorial analysis of transcriptomic datasets of CRPC cells (GSE13919, GSE99378, and GSE80743). **g** Bar plots show enriched canonical pathways for AR/AR-V7 signaling-independent HSF1-DBC1 co-targets and for HSF1-DBC1-AR/AR-V7 common targets in SM1 cells. **h** Pie chart showing numbers of downregulated, upregulated, and unchanged HSF1 ChIP-seq signals in SM1 versus SM1-D1KO cells. **i** Scatter plots showing changes in HSF1 ChIP-seq signals in 22RV1 versus D1KO cells under NHS and HS conditions. Cut-off: log2FC > 0.58 and P < 0.05 by MAnorm. **j** Venn diagram showing overlap between ChIP-seq peaks for DBC1 and HSF1 in SM1 cells.

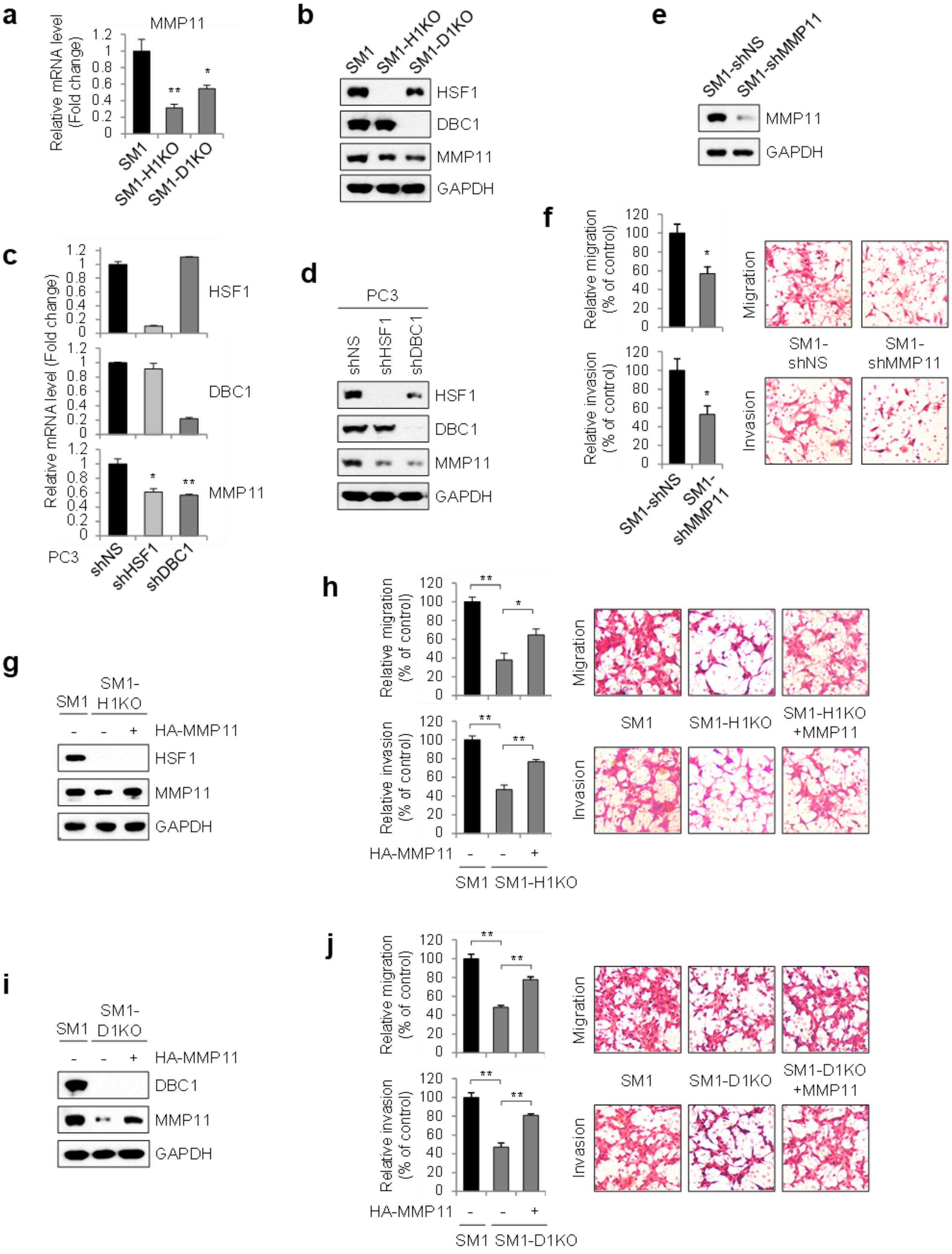
a
MMP11
Relative mRNA level (Fold change)
SM1
SM1-H1KO
SM1-D1KO
b
SM1
SM1-H1KO
SM1-D1KO
HSF1
DBC1
MMP11
GAPDH
e
SM1-shNS
SM1-shMMP11
MMP11
GAPDH
c
Relative mRNA level (Fold change)
HSF1
DBC1
MMP11
PC3
shNS
shHSF1
shDBC1
d
PC3
shNS
shHSF1
shDBC1
HSF1
DBC1
MMP11
GAPDH
f
Relative migration (% of control)
Relative invasion (% of control)
Migration
Invasion
SM1-shNS
SM1-shMMP11
g
SM1
SM1-H1KO
HA-MMP11
HSF1
MMP11
GAPDH
h
Relative migration (% of control)
Relative invasion (% of control)
HA-MMP11
SM1
SM1-H1KO
Migration
Invasion
SM1
SM1-H1KO
SM1-H1KO +MMP11
i
SM1
SM1-D1KO
HA-MMP11
DBC1
MMP11
GAPDH
j
Relative migration (% of control)
Relative invasion (% of control)
HA-MMP11
SM1
SM1-D1KO
Migration
Invasion
SM1
SM1-D1KO
SM1-D1KO +MMP11

Supplementary Fig. 7. MMP11 is a key downstream target of HSF1-DBC1 axis in mCRPC cells. a qRT-PCR analyses of MMP11 gene in SM1, SM1-H1KO, and SM1-D1KO cells. Data are means ± s.d. (n = 3). *P < 0.01 and **P < 0.001. **b** Protein levels were monitored in SM1, SM1-H1KO, and SM1-D1KO cells by immunoblot using indicated antibodies. **c** qRT-PCR analyses of HSF1, DBC1, and MMP11 genes in PC3-shNS, PC3-shHSF1, and PC3-shDBC1 cells. Data are means ± s.d. (n = 3). *P < 0.01 and **P < 0.001. **d** Protein levels were monitored in PC3-shNS, PC3-shHSF1, and PC3-shDBC1 cells by immunoblot using indicated antibodies. **e** Protein levels were analyzed in SM1-shNS and SM1-shMMP11 cells by immunoblot using indicated antibodies. **f** Transwell migration and invasion analyses of SM1-shNS and SM1-shMMP11 cells. *P < 0.01. **g** Cell lysates of SM1 and SM1-H1KO cells transfected with empty or HA-MMP11 expression vector were immunoblotted with indicated antibodies. **h** Transwell migration and invasion analyses of SM1 and SM1-H1KO cells transfected with empty or HA-MMP11 expression vector. *P < 0.01 and **P < 0.001. **i** Cell lysates of SM1 and SM1-D1KO cells transfected with empty or HA-MMP11 expression vector were immunoblotted with indicated antibodies. **j** Transwell migration and invasion analyses of SM1 and SM1-D1KO cells transfected with empty or HA-MMP11 expression vector. **P < 0.001.

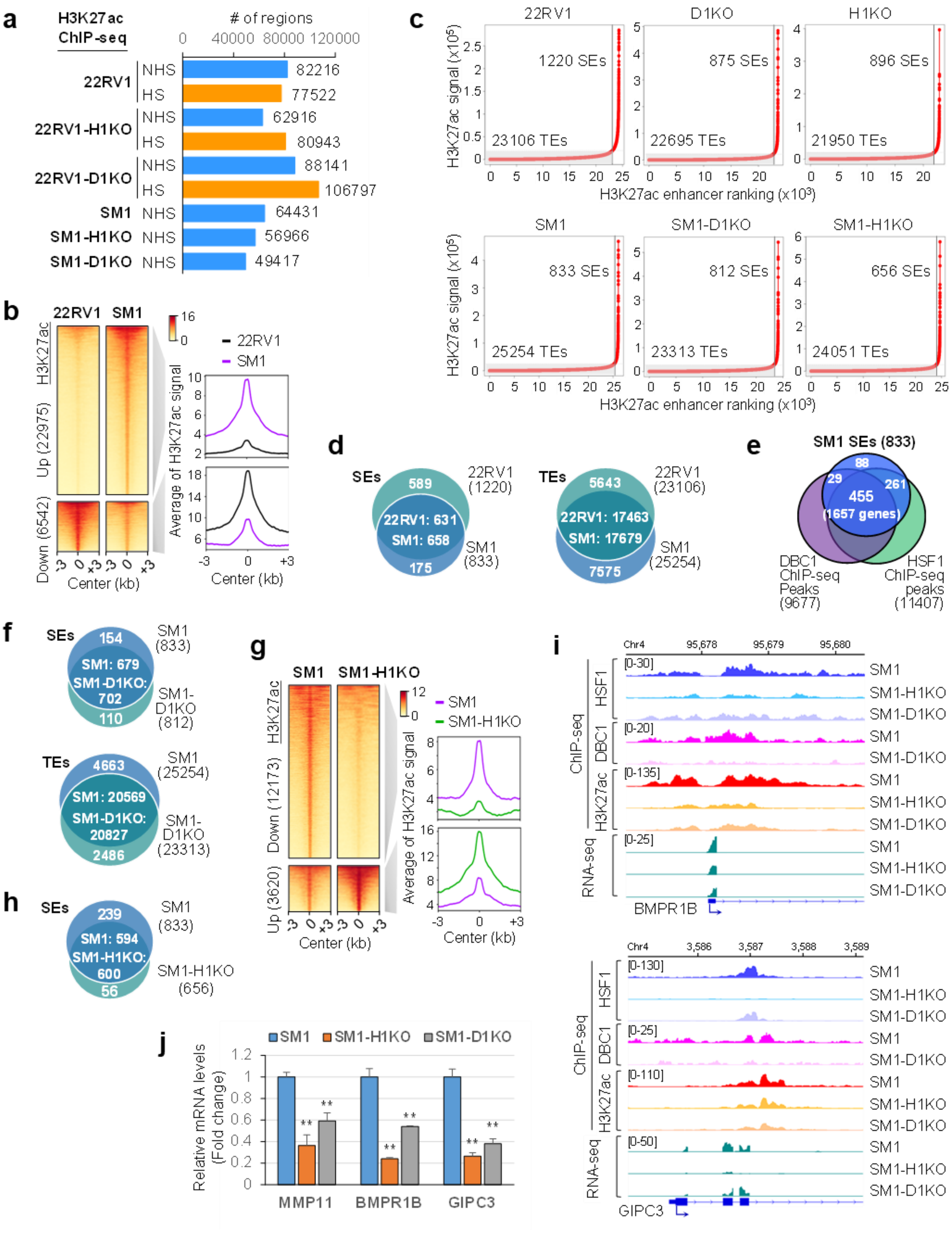

a
H3K27ac ChIP-seq
# of regions
0 40000 80000 120000
22RV1 NHS 82216
HS 77522
22RV1-H1KO NHS 62916
HS 80943
22RV1-D1KO NHS 88141
HS 106797
SM1 NHS 64431
SM1-H1KO NHS 56966
SM1-D1KO NHS 49417
b
22RV1 SM1
H3K27ac
Up (22975)
Down (6542)
Center (kb)
22RV1
SM1
Average of H3K27ac signal
c
22RV1 D1KO H1KO
1220 SEs 875 SEs 896 SEs
23106 TEs 22695 TEs 21950 TEs
H3K27ac signal (x10^5)
H3K27ac enhancer ranking (x10^3)
SM1 SM1-D1KO SM1-H1KO
833 SEs 812 SEs 656 SEs
25254 TEs 23313 TEs 24051 TEs
d
SEs 589 22RV1 (1220) 22RV1: 631 SM1: 658 175 SM1 (833)
TEs 5643 22RV1 (23106) 22RV1: 17463 SM1: 17679 7575 SM1 (25254)
e
SM1 SEs (833)
88 29 261 455 (1657 genes)
DBC1 ChIP-seq Peaks (9677)
HSF1 ChIP-seq peaks (11407)
f
SEs 154 SM1 (833) SM1: 679 SM1-D1KO: 702 110 SM1-D1KO (812)
TEs 4663 SM1 (25254) SM1: 20569 SM1-D1KO: 20827 2486 SM1-D1KO (23313)
g
SM1 SM1-H1KO
H3K27ac
Down (12173)
Up (3620)
Center (kb)
SM1
SM1-H1KO
Average of H3K27ac signal
h
SEs 239 SM1 (833) SM1: 594 SM1-H1KO: 600 56 SM1-H1KO (656)
i
Chr4 95,678 95,679 95,680
ChIP-seq HSF1 DBC1 H3K27ac
RNA-seq
SM1 SM1-H1KO SM1-D1KO
BMPR1B
Chr4 3,586 3,587 3,588 3,589
GIPC3
j
SM1 SM1-H1KO SM1-D1KO
Relative mRNA levels (Fold change)
MMP11 BMPR1B GIPC3

Supplementary Fig. 8. DBC1 plays an important role in regulating the SE landscape.
a Numbers of H3K27ac ChIP-seq peaks in 22RV1 (NHS and HS), H1KO (NHS and HS), D1KO (NHS and HS), SM1 (NHS), SM1-H1KO (NHS), and SM1-D1KO (NHS) cells. **b** Heatmaps of upregulated and downregulated H3K27ac ChIP-seq signals in 22RV1 versus SM1 cells (left). Signals within 3 kb around the center of H3K27ac ChIP-seq peaks are ordered by decreasing ChIP-seq signal in SM1 cells. Plots of average H3K27ac ChIP-seq signals at upregulated and downregulated regions (right). **c** Ranked plots of SEs and TEs defined based on H3K27ac ChIP-seq signals in 22RV1, D1KO, H1KO, SM1, SM1-D1KO, and SM1-H1KO cells. **d** Venn diagrams show overlap of SEs or TEs identified in 22RV1 and SM1 cells. **e** Venn diagram showing overlap of SEs with DBC1 and HSF1 ChIP-seq peaks. A total of 455 SEs (1,657 SE-associated genes) in the overlap are identified as HSF1-DBC1-occupied SEs. **f** Venn diagrams show overlap of SEs or TEs identified in SM1 and SM1-D1KO cells. **g** Heatmaps of downregulated and upregulated H3K27ac ChIP-seq signals in SM1 versus SM1-H1KO cells (left). Plots of average H3K27ac ChIP-seq signals at downregulated and upregulated regions (right). **h** Venn diagram showing overlap of SEs identified in SM1 and SM1-H1KO cells. **i** Representative snapshots of ChIP-seq tracks for HSF1, DBC1, and H3K27ac and RNA-seq tracks at SEs of BMPR1B and GIPC3 genes in SM1, SM1-H1KO, and SM1-D1KO cells. **j** qRT-PCR analyses of lost SE-associated genes in SM1, SM1-H1KO, and SM1-D1KO cells. Data are means ± s.d. ($n = 3$). **$P < 0.002$.

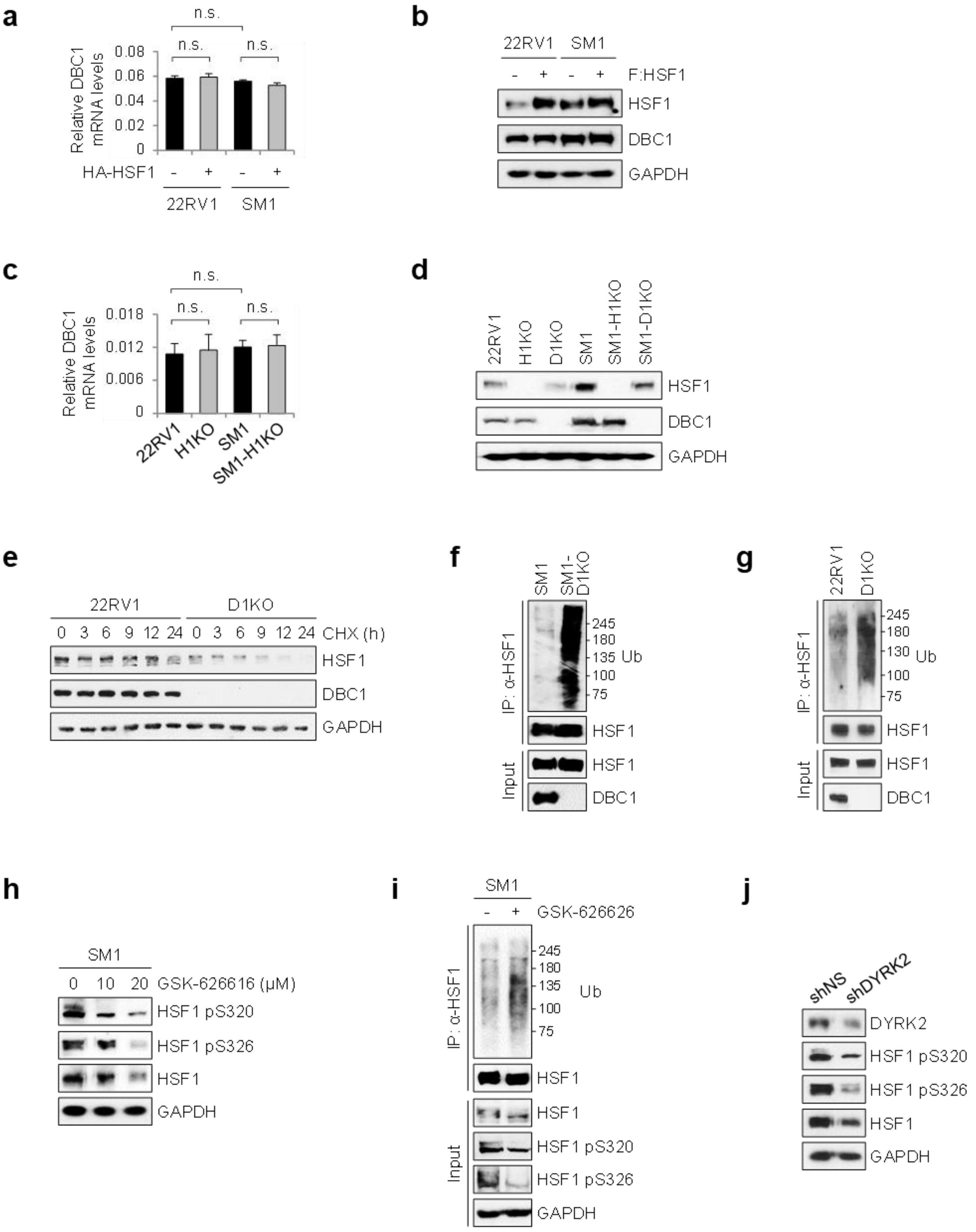
a
n.s.
n.s.
n.s.
Relative DBC1 mRNA levels
0.08
0.06
0.04
0.02
0
HA-HSF1 - + - +
22RV1
SM1
b
22RV1 SM1
- + - + F:HSF1
HSF1
DBC1
GAPDH
c
n.s.
n.s.
n.s.
Relative DBC1 mRNA levels
0.018
0.012
0.006
0
22RV1
H1KO
SM1
SM1-H1KO
d
22RV1
H1KO
D1KO
SM1
SM1-H1KO
SM1-D1KO
HSF1
DBC1
GAPDH
e
22RV1 D1KO
0 3 6 9 12 24 0 3 6 9 12 24 CHX (h)
HSF1
DBC1
GAPDH
f
SM1
SM1-D1KO
IP: α-HSF1
245
180
135 Ub
100
75
HSF1
Input
HSF1
DBC1
g
22RV1
D1KO
IP: α-HSF1
245
180
130 Ub
100
75
HSF1
Input
HSF1
DBC1
h
SM1
0 10 20 GSK-626616 (μM)
HSF1 pS320
HSF1 pS326
HSF1
GAPDH
i
SM1
- + GSK-626626
IP: α-HSF1
245
180
135
100
75
Ub
HSF1
Input
HSF1
HSF1 pS320
HSF1 pS326
GAPDH
j
shNS
shDYRK2
DYRK2
HSF1 pS320
HSF1 pS326
HSF1
GAPDH

Supplementary Fig. 9. DBC1 enhances HSF1 stability by regulating the phosphorylation and ubiquitination of HSF1.

a mRNA levels of DBC1 were analyzed by qRT–PCR in 22RV1 and SM1 cells transfected with empty or HA-HSF1 expression vector. Data are means ± s.d. (n = 3). n.s., not significant (P > 0.05). **b** 22RV1 and SM1 cell lysates transfected with empty or HA-HSF1 expression vector were immunoblotted with indicated antibodies. **c** mRNA levels of DBC1 were analyzed by qRT–PCR in 22RV1, H1KO, SM1, and SM1-H1KO cells. Data are means ± s.d. (n = 3). n.s., not significant (P > 0.05). **d** Cell lysates of 22RV1, SM1, and their H1KO and D1KO counterparts were analyzed by immunoblot with indicated antibodies. **e** 22RV1 and D1KO cells were treated with 50 μg/ml cycloheximide (CHX) and harvested at the indicated time. Cell lysates were analyzed by immunoblot with indicated antibodies. **f-g** Cell lysates of SM1 and SM1-D1KO cells (f) or 22RV1 and D1KO cells (g) treated with 20 μM MG132 for 4 h were immunoprecipitated with anti-HSF1 antibody and immunoblotted with indicated antibodies. **h** SM1 cells were treated with indicated concentrations of GSK-626616 for 24 h, and cell lysates were analyzed by immunoblot with indicated antibodies. **i** SM1 cell lysates treated with or without 20 μM GSK-626616 for 12 h and with 20 μM MG132 for 5 h (IP samples only) were immunoprecipitated with anti-HSF1 antibody and analyzed by immunoblot with indicated antibodies. **j** SM1 cells were infected with lentiviruses expressing a shRNA targeting DYRK2 (shDYRK2) or a non-specific shRNA control (shNS). Cell lysates were analyzed by immunoblot with indicated antibodies.

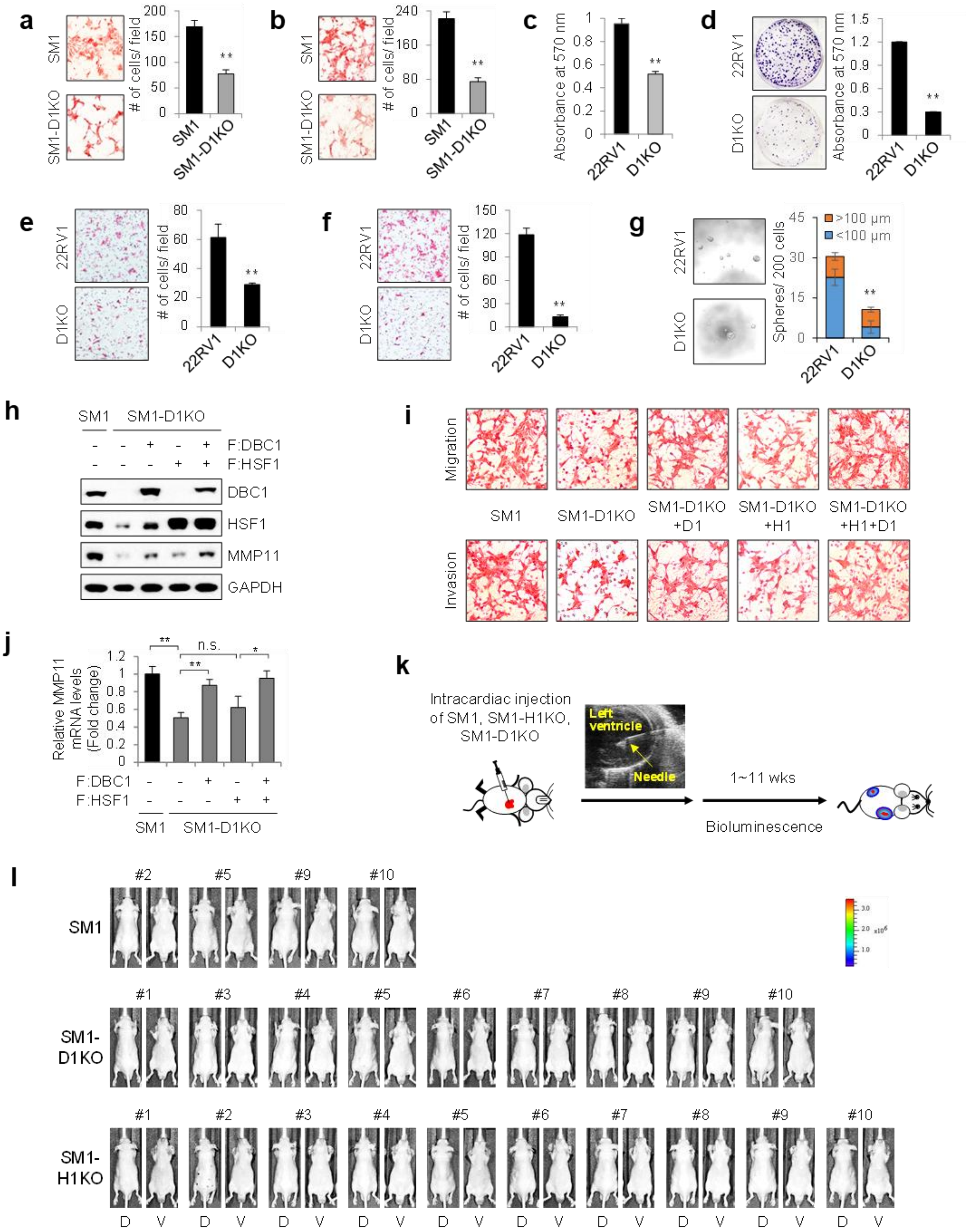

a
SM1
SM1-D1KO
# of cells/ field
200
150
100
50
0
**
SM1
SM1-D1KO
b
SM1
SM1-D1KO
# of cells/ field
240
160
80
0
**
SM1
SM1-D1KO
c
Absorbance at 570 nm
1
0.8
0.6
0.4
0.2
0
**
22RV1
D1KO
d
22RV1
D1KO
Absorbance at 570 nm
1.5
1.2
0.9
0.6
0.3
0
**
22RV1
D1KO
e
22RV1
D1KO
# of cells/ field
80
60
40
20
0
**
22RV1
D1KO
f
22RV1
D1KO
# of cells/ field
150
120
90
60
30
0
**
22RV1
D1KO
g
22RV1
D1KO
Spheres/ 200 cells
45
30
15
0
>100 μm
<100 μm
**
22RV1
D1KO
h
SM1
SM1-D1KO
F:DBC1
F:HSF1
DBC1
HSF1
MMP11
GAPDH
i
Migration
Invasion
SM1
SM1-D1KO
SM1-D1KO +D1
SM1-D1KO +H1
SM1-D1KO +H1+D1
j
Relative MMP11 mRNA levels (Fold change)
1.2
1
0.8
0.6
0.4
0.2
0
**
n.s.
**
*
F:DBC1
F:HSF1
SM1
SM1-D1KO
k
Intracardiac injection of SM1, SM1-H1KO, SM1-D1KO
Left ventricle
Needle
1~11 wks
Bioluminescence
l
SM1
#2
#5
#9
#10
SM1- D1KO
#1
#3
#4
#5
#6
#7
#8
#9
#10
SM1- H1KO
#1
#2
#3
#4
#5
#6
#7
#8
#9
#10
D
V

Supplementary Fig. 10. DBC1 and HSF1 are required for the tumorigenic and metastatic potential of mCRPC cells.

a-b Transwell migration (a) and invasion analyses (b) of SM1 and SM1-D1KO cells. **P < 0.001. **c-g** Cell proliferation (c), colony formation (d), migration (e), invasion (f), and sphere formation analysis (g) of 22RV1 and D1KO cells. **P < 0.001. **h** Cell lysates of SM1 and SM1-D1KO cells transfected with FLAG-HSF1 or/and FLAG-DBC1 expression vector were analyzed by immunoblots with the indicated antibodies. **i** Transwell migration and invasion analyses of SM1 and SM1-D1KO cells transfected with FLAG-HSF1 or/and FLAG-DBC1 expression vector. **j** mRNA levels of MMP11 were analyzed by qRT–PCR in SM1 and SM1-D1KO cells transfected with FLAG-HSF1 or/and FLAG-DBC1 expression vector. Data are means ± s.d. (n = 3). **P < 0.01 and *P < 0.05. n.s., not significant (P > 0.05). **k** Schematic diagram of a mouse metastasis model of SM1, SM1-H1KO, and SM1-D1KO cells. SM1, SM1-H1KO, and SM1-D1KO cells were intracardiacally injected into the left cardiac ventricle of 6-week-old male nude mice guided by ultrasound (n = 10 per group). Bioluminescence imaging (dorsal and ventral sides) was performed weekly for 11 weeks to monitor tumor metastasis. **l** Bioluminescence images of metastasis-free mice at day 78.

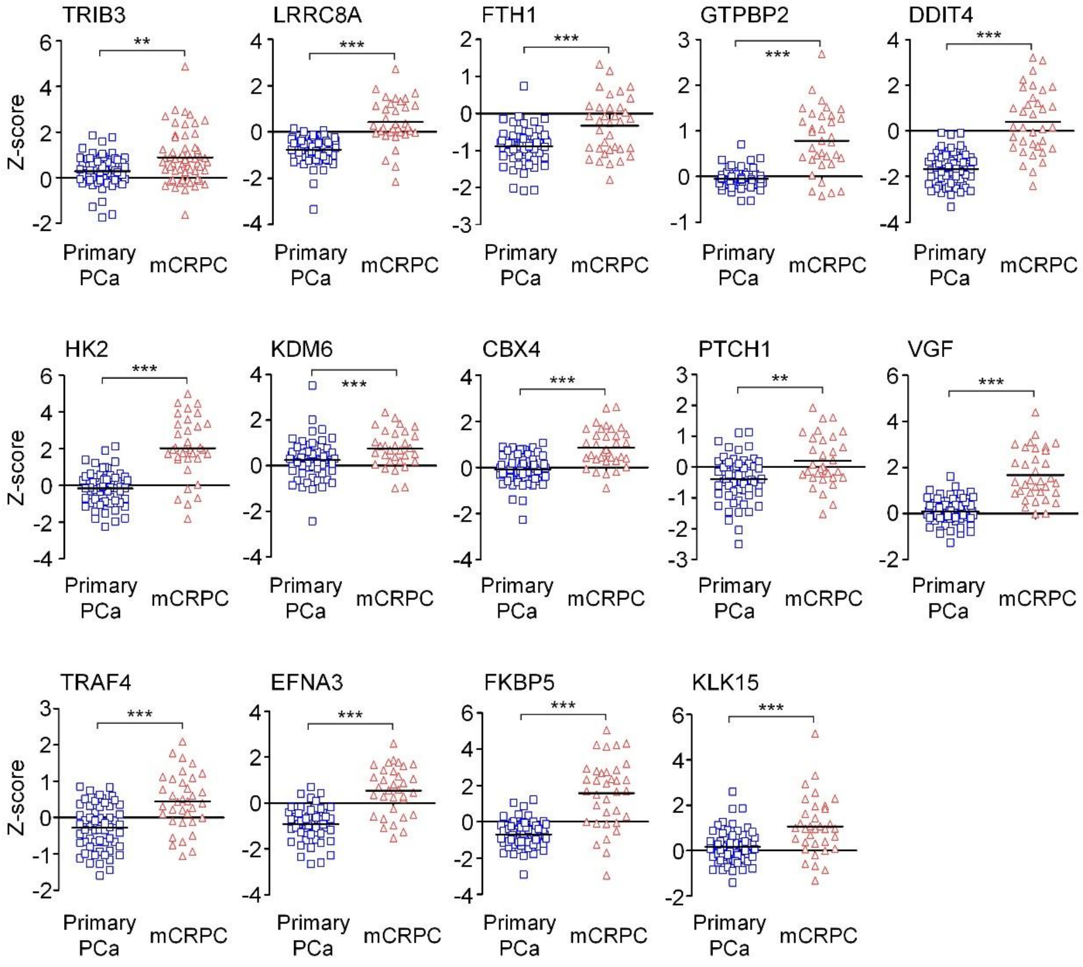

Supplementary Fig. 11. Metastasis-associated HSF1-DBC1 direct co-target genes were upregulated in mCRPC compared to primary PCa patients.

Transcript levels of metastasis-associated HSF1-DBC1 direct co-target genes from the GSE35988 dataset were determined in primary PCa and mCRPC. **P < 0.001, ***P < 0.0001.

Supplementary Table 1. Primer sets and oligonucleotides used in this study

sgRNA oligonucleotides

sgRNA.HSF1#1	Sense : CAC CGG TGG ACC CTC GTG AGC GAC C<br>Anti-sense : AAA CGG TCG CTC ACG AGG GTC CAC C
sgRNA.DBC1#1	Sense : CAC CGG GAG TGA GCA AAC CAG GAG G<br>Anti-sense : AAA CCC TCC TGG TTT GCT CAC TCC C
sgRNA.DBC1#3	Sense : CAC CGG GCA TTC TGG GAC AGT TCT G<br>Anti-sense : AAA CCA GAA CTG TCC CAG AAT GCC C

shRNA oligonucleotides

shDYRK2#3	Sense : CCG GGC AGG ACA AGG ATA ACA CAA TCT CGA GAT TGT GTT ATC CTT GTC CTG CTT TTT G<br>Anti-sense : AAT TCA AAA AGC AGG ACA AGG ATA ACA CAA TCT CGA GAT TGT GTT ATC CTT GTC CTG C
shHSF1#1	Sense : CCG GGC AGG TTG TTC ATA GTC AGA ACT CGA GTT CTG ACT ATG AAC AAC CTG CTT TTT G<br>Anti-sense : AAT TCA AAA AGC AGG TTG TTC ATA GTC AGA ACT CGA GTT CTG ACT ATG AAC AAC CTG C
shMMP11#1	Sense : CCG GCG CCT TCT ACA CCT TTC GCT ACT CGA GTA GCG AAA GGT GTA GAA GGC GTT TTT G<br>Anti-sense : AAT TCA AAA ACG CCT TCT ACA CCT TTC GCT ACT CGA GTA GCG AAA GGT GTA GAA GGC G

Primers used for qRT-PCR

HSF1 qRT-PCR	Forward : GAA CAG CTT CCA CGT GTT CGA<br>Reverse : TCG ATG TGG ACC ACT TTC CGG
MMP11 qRT-PCR	Forward : ATG ACT GCA GGG GCG TTC AA<br>Reverse : CAA AGG AGG CCT CAC AGG CA
BMPR1B qRT-PCR	Forward : TCC ACC ACC CTA GAC GCT AA<br>Reverse : CCC AGG TCA GCA ATA CAG CA
GIPC3 qRT-PCR	Forward : AGA CGT CCA AGA AGA CAG CG<br>Reverse : AAC TAG CCA CAG GCC TCT CT
DBC1 qRT-PCR	Forward : AAG GTG CAA ACG CTC TCC AAC CAG<br>Reverse : GGA TGT TTG GAA GAG ACT CAG AG
β-actin qRT-PCR	Forward : CCA CAC TGT GCC CAT CTA CG<br>Reverse : AGG ATC TTC ATG AGG TAG TCA GTC AG
GAPDH qRT-PCR	Forward : TCT GGT AAA GTG GAT ATT GTT GCC<br>Reverse : GAA GAT GGT GAT GGG ATT TCC

SUPPLEMENTARY REFERENCES

1. Moon, S.J. *et al.* Bruceantin targets HSP90 to overcome resistance to hormone therapy in castration-resistant prostate cancer. *Theranostics* **11**, 958-973 (2021).
2. Yu, E.J. *et al.* Reciprocal roles of DBC1 and SIRT1 in regulating estrogen receptor alpha activity and co-activator synergy. *Nucleic Acids Res* **39**, 6932-6943 (2011).
3. Kim, J.H. *et al.* CCAR1, a key regulator of mediator complex recruitment to nuclear receptor transcription complexes. *Mol Cell* **31**, 510-519 (2008).
4. Moon, S.J. *et al.* DBC1 promotes castration-resistant prostate cancer by positively regulating DNA binding and stability of AR-V7. *Oncogene* **37**, 1326-1339 (2018).
5. Kim, H.J., Moon, S.J., Hong, S., Won, H.H. & Kim, J.H. DBC1 is a key positive regulator of enhancer epigenomic writers KMT2D and p300. *Nucleic Acids Res* **50**, 7873-7888 (2022).
6. Kim, H.J., Kim, S.H., Yu, E.J., Seo, W.Y. & Kim, J.H. A positive role of DBC1 in PEA3-mediated progression of estrogen receptor-negative breast cancer. *Oncogene* **34**, 4500-4508 (2015).
7. Yu, E.J. *et al.* Positive regulation of beta-catenin-PROX1 signaling axis by DBC1 in colon cancer progression. *Oncogene* **35**, 3410-3418 (2016).
8. Whyte, W.A. *et al.* Master transcription factors and mediator establish super-enhancers at key cell identity genes. *Cell* **153**, 307-319 (2013).
9. Prince, T.L. *et al.* Client Proteins and Small Molecule Inhibitors Display Distinct Binding Preferences for Constitutive and Stress-Induced HSP90 Isoforms and Their Conformationally Restricted Mutants. *PLoS One* **10**, e0141786 (2015).
